# SARS-CoV-2 Vaccine-Induced Seroconversion and Immune Correlates in Patients with Hematological Malignancies. A Real World Study

**DOI:** 10.32604/or.2025.067561

**Published:** 2025-09-26

**Authors:** Norbert Nass, Mohamad-Kamal Yaakoub, Alexandra-Victorita Simion, Hartmut Kroll, Sabine Westphal, Judith Pannier, Gerhard Behre

**Affiliations:** 1 Department of Internal Medicine 1, Dessau Medical Centre and Brandenburg Medical School Theodor-Fontane, Dessau, 06847, Germany; 2 Institute of Pathology, Medical School Brandenburg Theodor-Fontane, University Hospital Brandenburg/Havel, Brandenburg, 14770, Germany; 3 Institute of Clinical Chemistry, Dessau Medical Centre and Brandenburg Medical School Theodor-Fontane, Dessau, 06847, Germany; 4 Institute for Transfusion Medicine Dessau, Red Cross Blood Transfusion Service NSTOB, Dessau, 06847, Germany

**Keywords:** Severe Acute Respiratory Syndrome Coronavirus 2 vaccination, hematologic malignancies, lymphoma, myeloma, seroconversion

## Abstract

**Background:**

Patients with hemato-oncological malignancies may respond insufficiently to vaccination, especially in terms of antibody titer. The antibody response depends on the type of malignancy as well as the type and timing of treatment. We intended to evaluate this using real-world data from patients of our regional hospital. This study also considers the role of immune status, including T-cell activation markers, in predicting vaccination success.

**Methods:**

Seventeen patients of our hospital having a hematological malignancy were included in this study, including myeloma, lymphoma, as well as acute myeloid leukemia (AML) and chronic lymphoid leukemia (CLL). All patients were vaccinated against Severe Acute Respiratory Syndrome Coronavirus 2 (SARS-CoV-2) using Tozinameran following current recommendations. Circulating antibodies directed against the spike protein of SARS-CoV-2 were determined by a commercial immune assay. Immune status was determined from peripheral blood by flow cytometry. Both parameters were followed in fifteen patients who provided sufficient follow-up data for up to one year. Patients were categorized as responders or non-responders, and differences in diagnosis, treatment, and immune status were analyzed.

**Results:**

Antibody response depended on both diagnosis and treatment. Active treatment directed against B-cells, such as anti-Cluster of Differentiation 20 (CD20) therapy, was associated with weak seroconversion. For CD38-as well as proteasome-directed therapies, the data suggest that responders as well as non-responders exist. Notably, low peripheral B-cell numbers and high CD3+HLADR+cell counts correlated with weak seroconversion upon vaccination.

**Conclusions:**

We suggest that peripheral immune status can be applied as a predictive biomarker for seroconversion upon vaccinations.

## 1 Introduction

Vaccination against Severe Acute Respiratory Syndrome Coronavirus 2 (SARS-CoV-2) has been the most effective tool in preventing further spread of the virus [1]. However, not all patients develop adequate immune responses post-vaccination [2], particularly those with a compromised immune system [3], the elderly [4], and oncologic patients undergoing active treatment [5,6]. Among these vulnerable groups, patients with hematological malignancies are particularly at risk, as these malignancies target specifically cells of the immune system and their treatments further impair immune functionality [7–9].

Most patients with solid cancer respond sufficiently to vaccination in terms of seroconversion (94%–98%), whereas hemato-oncological patients exhibited only 85% or less [10–12]. Older adults seem to be underrepresented in vaccination studies [13]. Nevertheless, people over 60 years are reported in a meta study [14] to have a standard mean difference in response of about −2, compared to −3.66 for hematological malignancies and −0.85 for solid cancers. Especially Cluster of Differentiation 20 (CD20) directed treatment, which depletes B-cells, is of concern, and several strategies to overcome the apparent insufficient antibody response have been suggested. This includes improved timing of the vaccination, including monitoring of B-cell numbers [15,16]. In addition to neutralizing antibodies, T-cell responses play a critical role in providing protection against SARS-CoV-2 infection in cases where humoral immunity is insufficient [17]. Indeed, current vaccines stimulate significant T-cell responses that clearly contribute to their protective effect against COVID-19 [18,19]. Thus, antibody production as well as cellular immunity should be considered to obtain a complete picture of vaccination results [20]. Current research tries to define predictive markers for immunization failure in order to improve future vaccination strategies in vulnerable groups. By analyzing the immunome and not only focusing on humoral or cellular responses but also considering occurring infections, a promising cellular signature has been proposed recently [21].

Altogether, understanding predictors of vaccine response could enhance the effectiveness of vaccination programs for vulnerable patients [22]. Nevertheless, passive immunization using monoclonal antibodies has been proposed as an alternative for those who fail to respond to vaccination [23,24] and has shown promise in reducing severe COVID-19 outcomes [25,26].

This study focuses on real-world data from patients at the Dessau Medical Centre (Städtisches Klinikum Dessau), examining the relationship between vaccine response, diagnosis, and treatment in hemato-oncological patients. By evaluating antibody titers and immune status, we aim to identify parameters that could serve as predictive markers for vaccine-induced seroconversion in this population.

## 2 Materials and Methods

### 2.1 Patients

**Figure 1:**
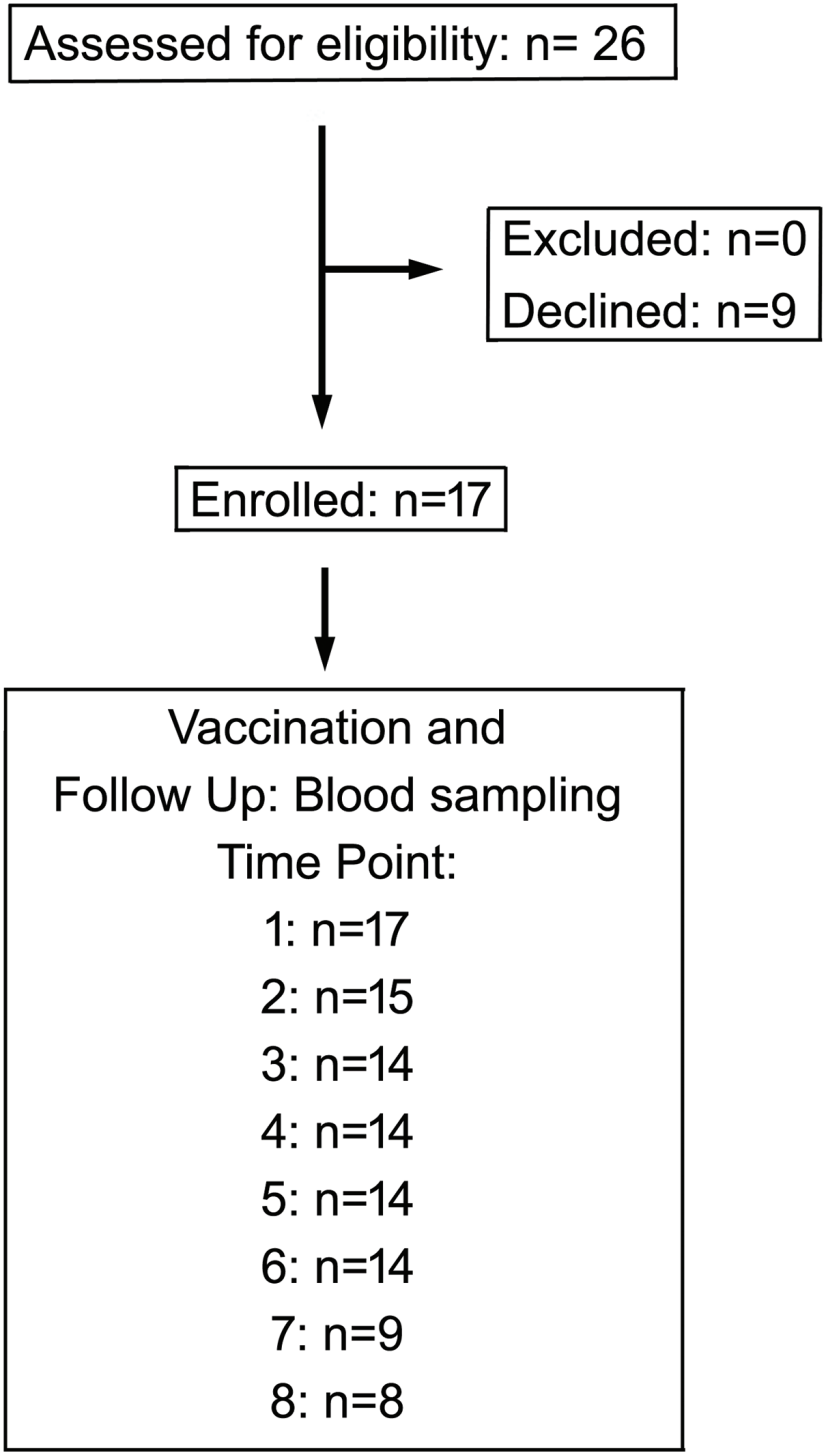
Flow chart of the study showing enrollment and participation of patients in this study

This study involved initially 26 patients with various hematologic malignancies diagnosed and treated at the Dessau Medical Centre from January to April 2021. The follow-up time was 1 year upon vaccination; the last blood samples were taken in April 2022. Diagnosis was done using the current guidelines of the European Society for Medical Oncology (www.esmo.org) for hemato-oncological malignancies and extracted from the patients’ records of the clinic. Patients aged under 18 were excluded, as well as patients with prior SARS-CoV-2 infection based on medical history and positive nucleocapsid antibody testing. The patients gave their consent to participate in this study; otherwise, they were excluded. The study has been approved by the Ethics Committee of the Medical Association of Saxony-Anhalt, Germany (record number #16/21) and was registered at the DKRS (German Clinical Trials Register) under the ID DRKS00024551. Of the initial 26 patients, 9 refused to participate. Due to non-compliance or inability to provide sufficient samples, patients gradually dropped out of the study, and only 15 could be included in the longitudinal statistical analysis. To the best of our knowledge, none of the patients contracted COVID-19 during the follow-up time. The median age was 63 (range: 42 to 87), and 7 (41%) female and 10 (59%) male patients were included in the final analysis. A flow diagram summarizing patient recruitment and dropout is provided in Fig. 1. All patients were vaccinated twice using the mRNA vaccine Tozinameran (Comirnaty, BioNTech/Pfizer) following the respective guidelines [27]. First vaccination was done in January 2021 (patients 1–7, Table 1) or April 2021 (patients 8–18, Table 1). The last blood samples were taken in April 2022. A parallel study investigated analogous patients analyzing T-cell responses to SARS-CoV-2 vaccination [27].

**Table 1:** Characterization of patients according to diagnosis and antibody response

Case number	Sex	Age	Diagnosis	Comorbidities	Medication preceding or during vaccination	Antibody response (iU) 4 weeks after first vaccination	Antibody response (iU) 8 weeks after first vaccination
***Weak responders***							
1	m	58	Mantle cell lymphoma		R-CHOP (2017), Ibrutinib (since 2.2019), Immunoglobulin therapy (since 11.2019)	Neg.	Neg.
2	w	73	Multiple myeloma	Hypertension, atrial fibrillation	CyBorD (Cyclophosphamide + Bortezomib + dexamethasone) (2019/2020), zoledronic acid (since 1.20)	313.3	164.6
5	m	56	Multiple myeloma	Steatosis hepatitis, COPD, hypertension, obesity	Bendamustine/Bortezomib (7-9.2020)	124.4	105.8
6	w	61	Follicular lymphoma		CHOP (2014), Rituximab (2015–2016), Rituximab (4.-9.2019), Rituximab (2020), Obi-CHOP (2021), Radiotherapy (2021)	23.8	n.d.
7	m	44	Follicular lymphoma	Testicular cancer (2001), chemotherapy	Obi-CHOP (5.2021)	Neg.	14.3
16	w	63	Multiple myeloma	Hypertension, hysterectomy	Daratumumab (06.2021)	Neg.	9.8
17	w	88	Follicular Lymphoma	Hypertension, heart valve insufficiency, atrial fibrillation kidney insufficiency hypothyreosis sarcoidosis	Preceding medication unknown, Rituximab (8.9.2022)	Neg.	Neg.
***Good responders***							
3	m	42	Multiple myeloma	Bronchial asthma	Bortezomib/ Lenalidomid (9.19–2.20), Transplantation, Lenalidomid (10.2020–1.2022),	2287	1516
4	m	58	CLL relapse	hypertension, microangiopathydiabetes	R-FC (Cyclo phosphamide-Fludarabine-Rituximab) (9.–11.2010 and 5.2017 to 9.2017), Ibrutinib (6.2018–11.2020)	456.4	819.4
8	m	66	AML		HD-Cytarabin (12.2020–1.2021), Cytarabin (2–3.2021)	Neg.	>2500
11	m	51	Myeloproliferative neoplasm (Thrombocytosis)	Apoplectic insult, cerebral microangiopathy hypertension	Imatinib (since 3.2019)	Neg.	757
12	w	72	Hypereosinophilic-syndrom		Since 3.2019 Imatinib, VCD (8.2019–1.2020), Lenalidomid (since 7.2020)	n.d.	n.d.
13	m	67	Multiple myeloma		VCD 08.2019, Transplantation 11.2019	n.d.	>2500
14	w	54	Non-hodgkin lymphoma		Daratumumab (5 12.2021), Daratumumab (3.2022)	24.22	2500
15	w	70	Multiple myeloma		Cisplatin/Etoposid (2.–6.2016), Topotecan (12.2016–4.2017), Pembrolizumab (3–8.2018), VCD (11.2019–3.2020), Daratumumab/Revlimid (10.2020–07.2021)	>2500	562
18	m	69	Multiple myeloma	Prostate-, renal-, thyroid-Carcinoma, hypertension, heart valve insufficiency, osteoporosis	Carfilzomib, Lenalidomid, Dexamethason (02.2021)	54.5	>2500

Note: n.d., not determined; Neg., below detection limit. Therapy dates are given in months.

### 2.2 Data Collection

Heparinized blood samples were drawn on the day of the first and second vaccination (two weeks after 1st), and then at weeks 2, 4, 8, 10, 12, 16, 32, 52 after the 2nd dose. Antibodies directed against the SARS-CoV-2 spike and nucleocapsid protein were determined using the Roche Elecsys S-Ab assay or the Roche Elecsys anti-nucleocapsid antibody (N-Ab) assay (Roche Diagnostics, Mannheim, Germany) in a Roche Cobas 6000 analysis system (Roche Diagnostics, Mannheim, Germany).

### 2.3 Flowcytometry

Fresh blood, anticoagulated with EDTA, was used for flow cytometry within 6 h after puncture. The CYTO-STAT tetraCHROME panel (6607073, Beckman Coulter, Krefeld, Germany) and antibodies for HLA-DR (B49180, Beckman Coulter, Krefeld, Germany) and IgG1 (A07795, Beckman Coulter, Krefeld, Germany) as isotope control were applied (antibody details are listed in Supplementary Table S3). This panel is certified for *in vitro* diagnostics (IVD) and used for our routine clinical diagnostic applications. Two 100 μL fractions of whole blood were stained with premixed five-color antibody cocktails for lymphocyte profiling, including B-cell subpopulations, CD4+ and CD8+ subpopulations, regulatory T-cells, and NK-cell activation markers. The first tube contained 10 μL each of CD45-FITC, CD56-PE, CD19-ECD, CD3-PC5, IgG1-PC7, and 20 μL of CD16-PE. A second tube contained CD45-FITC, CD4-PE, CD8-ECD, CD3-PC5, and HLA-DR-PC7. Erythrocyte lysis was achieved by adding the IMMUNOPREP Reagent System Whole Blood Lysing Reagent (7546999, Beckman Coulter, Krefeld, Germany). Samples were then measured by flow cytometry (Navios EX, Beckman Coulter, Krefeld, Germany). The CXP software (Beckman Coulter, Krefeld, Germany) was used for data analysis. Manufacturer guidelines were strictly followed for instrument calibration and quality control. A representative example for the determination of immune cell populations is shown in Supplementary Fig. S2.

### 2.4 Data Analysis

Data were analyzed using SPSS Statistics for Windows vers. 23 (IBM Corp., Armonk, NY, USA). Mann-Whitney-U test and one-way ANOVA were used for the comparison of experimental groups. For the determination of confidence intervals of contingency tables, R Studio vers. 2023.03.0 Build 386 for Windows was used. A *p* < 0.05 was considered significant; a *p* < 0.1 was interpreted as a statistical trend.

## 3 Results

### 3.1 Patient Characteristics and Seroconversion

**Figure 2:**
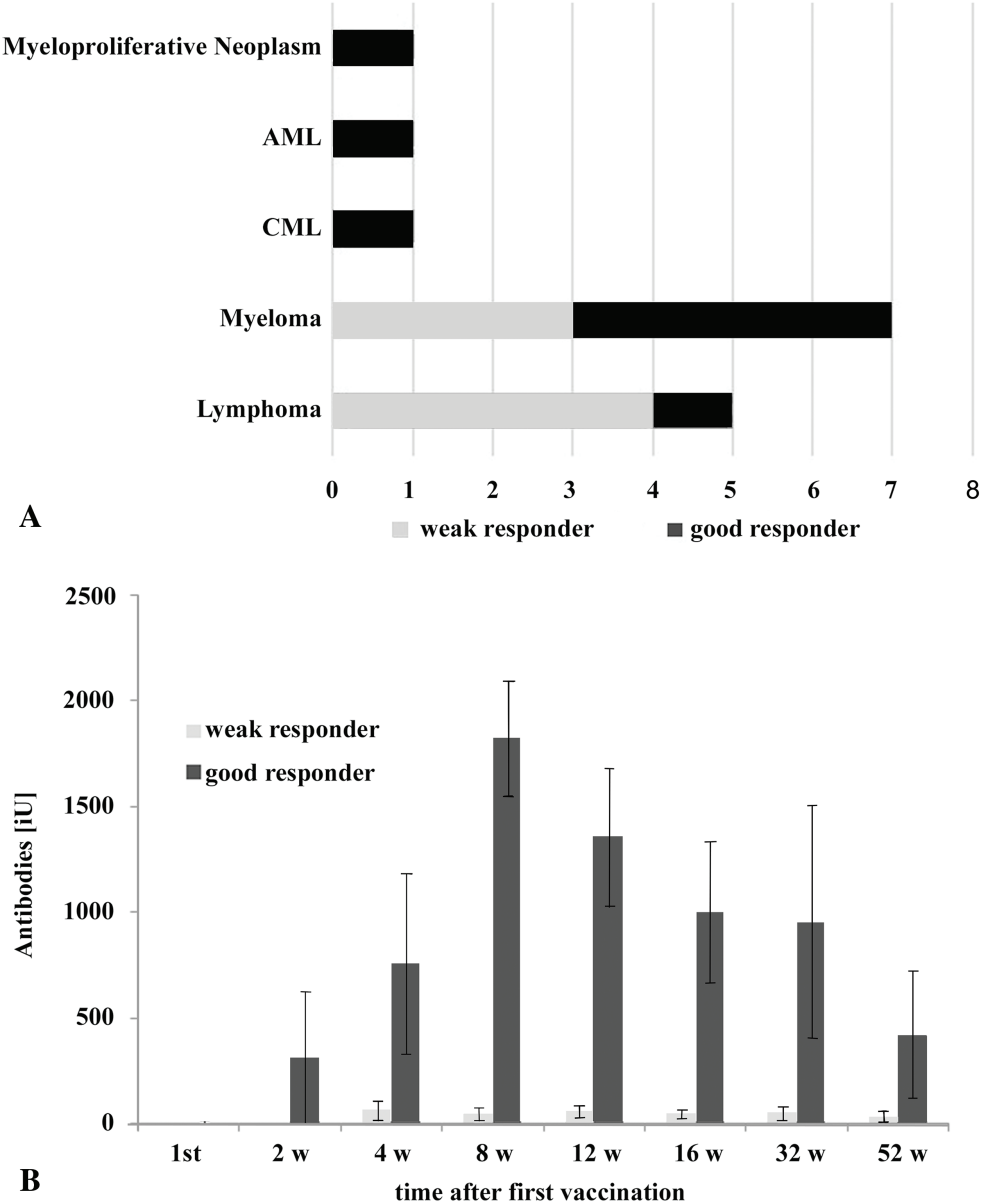
Seroconversion success in the vaccinated patients. (**A**): Representation of patients (15), showing diagnosis grouped in multiple myeloma (7), lymphoma (5), AML (1), CML (1), and myeloproliferative neoplasm (1). (**B**): Anti-SARS-CoV-2 Spike protein antibody titer in patients separated into good and weak responders. Average and standard deviation are shown. Antibody titers of the well-responding group were statistically significantly higher than in weak responders at time points after 4 weeks (ANOVA, *p* < 0.05). For detailed statistics, see also Supplementary Table S2

Patient diagnoses and antibody titers are shown in Table 1, and the complete dataset is provided in Supplementary Table S1. On average, the highest antibody titer was observed at eight weeks after immunization (Fig. 2B). Patients were defined as good responders when the antibody titer either at 4 or 8 weeks after vaccination was higher than 500 iU. This corresponds to the median antibody levels at these two time points (538 iU) and also to the 1st quartile of the seroconversion response of healthy subjects as determined in a parallel study (supplementary Fig. S1). Good responders were identified among multiple myeloma (4 of 7, 57%) and AML/CLL patients, and a patient suffering from myeloproliferative neoplasia responded to vaccination. However, only 1 of 5 (20%) lymphoma patients showed significant antibody levels (*p* = 0.298, 95% confidence interval = 0.003–3.943; contingency table: lymphoma/myeloma vs. responder/non-responder, Fisher’s exact test) (Fig. 2A, Table 1, Table S1).

### 3.2 Impact of Treatment

Treatment regimens significantly correlated with vaccine response. Non-responders included lymphoma patients receiving anti-CD20 monoclonal antibodies (Rituximab or Obinutuzumab) combined with the CHOP chemotherapy protocol. Among myeloma patients, 2 receiving bortezomib failed to respond, while 4 others on similar regimens showed robust antibody production. Interestingly, 2 of 3 patients treated with the CD38-targeting antibody daratumumab responded well to vaccination.

### 3.3 Immune Status as a Predictor

**Figure 3:**
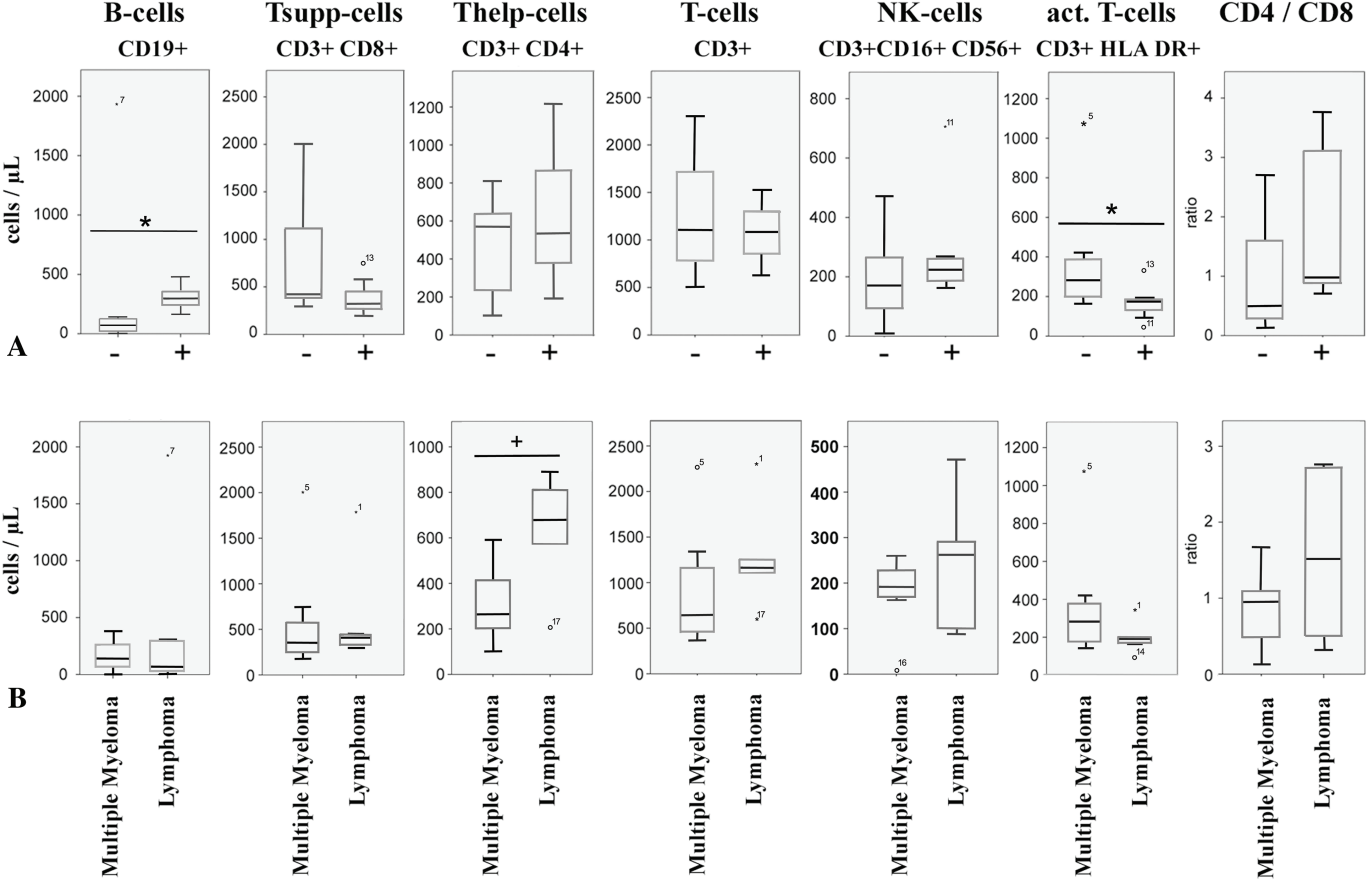
Peripheral immune status from patients included in this study. (**A**): Immune cell subtypes were determined by flow cytometry in blood taken before vaccination. − indicates weak response; + indicates good response to vaccination. ° and numbers indicate outliers, corresponding to Table 1 and the Supplementary Table S1, * indicates *p* < 0.05 (Mann-Whitney U-Test). (**B**): Peripheral immune cell counts in patients before vaccination. Patients were separated into cases of multiple myeloma and lymphoma. ° and numbers indicate outliers, corresponding to Table 1 and the Supplementary Table S1. * indicates *p* < 0.05, + indicates *p* < 0.1, Mann-Whitney U-Test

**Figure 4:**
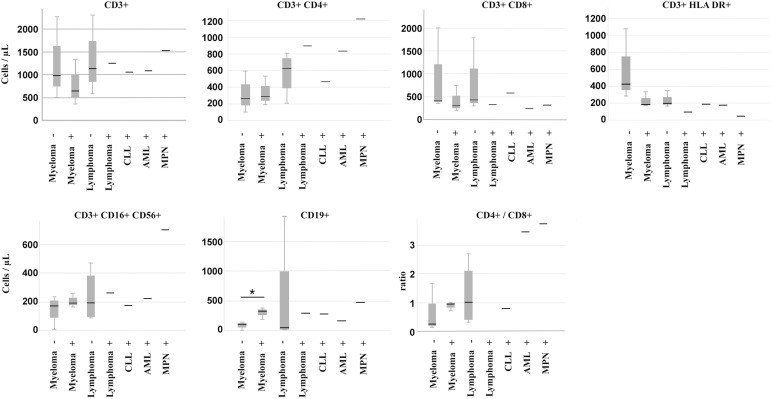
Peripheral immune cell numbers in patients separated according to diagnosis and vaccination response. −: weak response, +: good response, CLL: chronic lymphocytic leukemia, AML: acute myeloid leukemia, MPN: myeloproliferative neoplasia. * indicates *p* < 0.05, Mann-Whitney U-Test

Peripheral immune profiling revealed that responders had significantly higher B-cell counts (Median: 78/μL vs. 298/μL, *p* = 0.026, Mann-Whitney U-test) and lower activated T-cell (CD3+ HLA-DR+) counts (Median: 175/μL vs. 282/μL, *p* = 0.035, Mann-Whitney U-test) compared to non-responders (Fig. 3A) before vaccination. Lower B-cell counts were associated with active anti-CD20 therapy in lymphomas. Within lymphomas, the single responder exhibited higher CD4+ T-helper cell counts and a high CD4/CD8 ratio, while non-responders showed elevated activated T-cell counts. Lymphoma patients, who received mostly (4 out of 5) CD20-directed therapy, had, in general, higher numbers of T-helper cells (median: 679/μL vs. 265/μL, *p* = 0.062, Mann-Whitney U-test) (Fig. 3B) than myeloma patients (Supplementary Table S4). Myeloma responders also had higher B-cell counts and lower T-cell activation markers compared to non-responders (Fig. 4). Separate statistical analyses for the entities (Mann-Whitney U test) revealed little significance, presumably due to the low number of cases, except for myeloma patients (B-cells/μL; *p* = 0.049) (Table S5).

## 4 Discussion

Predicting the vaccination success, especially antibody production, has been the focus of several studies on hemato-oncological patients [28–30], lymphatic leukemias [31–33] or myeloma [34–36] as a deeper understanding has the potential to further improve the protection of such patients. This study highlights again the influence of diagnosis and treatment on SARS-CoV-2 vaccine responses in patients with hematological malignancies. We analyzed hematological patients from the Dessau Medical Center (Städtisches Klinikum Dessau) with respect to the ability to produce S-protein-directed antibodies after SARS-CoV-2 vaccination and also determined their immune status before and after vaccination.

Compliance with the study protocol turned out to be limited, especially at later time points. We can only speculate on the reasons. Besides the urban areas of Dessau, our hospital covers rural areas with limited public transport facilities, especially for the elderly. At the time this study was conducted, there was also a significant risk of being infected by SARS-CoV-2 in public. Under such circumstances, patients’ motivation to visit the hospital for a study without direct relevance to their treatment seemed to be low. Thus, we suspect that especially elderly and multimorbid patients might be underrepresented in this study. The resulting small sample size and patient attrition represent key limitations of this study, restricting its generalizability. Future research and meta-studies involving larger, more diverse cohorts are essential to validate these findings and refine predictive biomarkers for vaccine response.

We were able to separate seven responders from eight non-responders based on antibody levels at 4 and 8 weeks after vaccination using a cut-off of about 500 iU. Within these two groups, we aimed at defining diagnosis, treatments, and immune cell status as predictors for the production of circulating anti-SARS-CoV-2 antibodies.

Within the lymphoma group, the percentage of responders was lower than in the myeloma patients (Fig. 1A). However, most probably due to the low number of patients, this was not statistically significant. B-cell lymphomas represent a diverse group of malignancies [37] exhibiting a compromised immune system [38]. For instance, the appearance of incompetent B-lymphocytes in CLL has been shown [39], as well as alterations in defined CD8+ subsets in follicular lymphoma [40]. For diffuse large B-cell lymphoma (DLBCL), a reduction of competent B cells and communication between B cells and other immune cells has been described [41]. Furthermore, lymphomas are mostly treated by targeting B-cells. As anticipated, such therapies using anti-CD20 antibodies were associated with poor seroconversion in our study. This aligns with prior research demonstrating prolonged immune suppression in patients treated with Rituximab or Obinutuzumab. An observation that was also observed for other diseases, which are subject to B-cell depletion therapy, such as multiple sclerosis [42] or vasculitis [43]. Such anti-CD20 therapies profoundly deplete B-cell populations, consistent with the weak seroconversion in treated lymphoma patients [44]. *Vice versa*, antibody response to SARS-CoV-2 vaccination depended on the B-cell reconstitution and, thus, time after the last rituximab treatment [45].

Consequently, low B-cell counts correlated significantly with the weak-responding phenotype in 4 out of 5 lymphoma cases in our study. The one well-responding lymphoma patient, however, exhibited especially high numbers of CD3+/CD4+ helper T-cell- and low CD3+/HLA-DR+ activated T-cell numbers. T-helper cells, especially follicular helper T-cells (Tfh-cells), have the capacity to improve antibody production of B-cells [46,47]. Nevertheless, significant antibody levels in this patient were delayed to the 8-week post-vaccination time point. Thus, this patient represents an exception in the group of lymphomas.

We also observed non-responding myeloma patients treated with proteasome inhibitors (Bortezomib, Carfilzomib). Two myeloma patients receiving Bortezomib did not respond to vaccination (Table 1), whereas 4 patients treated with proteasome inhibitor-containing therapy schemes showed significant seroconversion. These inconsistent responses among myeloma patients suggest that other factors, such as treatment timing and immune microenvironment, may modulate vaccine efficacy.

CD38-directed treatment of myeloma is described to inhibit antibody production after vaccination [48]. In our study, two patients were treated with the CD38-targeting antibody Daratumumab and exhibited sufficient antibody production; nevertheless, there was one Daratumumab-treated lymphoma patient who did not respond to the vaccination. We suspect that either the timing of the treatments or the particular disease may cause this difference, but again, our sample size is too low to draw distinct conclusions.

Immune dysregulation is a common feature in myelomas. Especially, the T-cell repertoire in bone marrow is shifted towards an exhaustion and suppressor phenotype, causing a reduced response to vaccinations [49]. In our study, myeloma patients responded more frequently to vaccination than lymphoma patients, and peripheral T-suppressor cell counts were similar to those of lymphoma patients and were within the reference range. However, the amount of tumor-infiltrating cells does not necessarily correspond to circulating cell numbers. In our study, weakly responding myeloma patients had high numbers of CD3+/CD4+ T-helper cells and showed no differences in CD3+/HLA DR+ cell numbers. Whether this causes the weak response to vaccination requires further studies and underscores the potential value of peripheral immune profiling as a predictive biomarker. In light of new data on the immunolome and a specific immune cell signature in non-responding cases, a more detailed determination of peripheral immune cells can improve this predictive value even further [22].

In a parallel study [27], the T-cell response upon vaccination of 12 patients with hemato-oncological malignancies was compared with the response of healthy subjects. Although not all of these patients produced antibodies against SARS-CoV-2, a robust T-cell response comparable to that of healthy subjects was detected. Interestingly, the hemato-oncological patients seemed to develop more SARS-CoV-2-specific T-cells (CD4+ and Tfh cells) than the control subjects. Also, active therapy appeared to correlate with a higher T-cell response rate. Similar to our study, focusing only on antibody responses, differences between lymphoma and myeloma patients were found, such as a higher frequency of SARS-CoV-2-specific Tfh-cells in myeloma patients. We also report here that myeloma patients responded better in terms of seroconversion and hypothesize that this is a consequence of B-cell-directed therapy in lymphoma patients. Taken these two studies together, active therapy, especially targeting B-cells, reduced seroconversion rates, but might have a positive influence on T-cell responses. Clearly, both responses contribute to resistance to the virus, and it is not clear which response contributes most to effective protection and the resolution of the disease. Nevertheless, the elevated T-cell responses reduce the risk of developing disease [50,51] even in the absence of seroconversion. Still, antibodies are an effective part of resistance, and efficient strategies to improve seroconversion rates are therefore necessary [15].

## 5 Clinical Implications

The vaccination of hemato-oncological patients, often under B-cell depleting therapy, remains a challenge, not only for SARS-CoV-2 but also for novel upcoming infectious diseases. Our data further underline that immune-profiling seems valuable for the prediction of efficient seroconversion [15]. This seems to us more valid than applying a general time scheme, as B-cell repopulation time after treatment varies considerably [52]. One might also consider different vaccination strategies for B-cell-depleted patients. Additional booster injections or different types of vaccines, either mRNA- or adeno-virus-based or combined approaches, might exhibit improved efficiencies in such cases. Indeed, in a cohort of B-cell-depleted patients with renal autoimmune disease, there were differences between the mRNA vaccines, with mRNA-1273 (Moderna) being more effective than BNT162b2 (BioNtech/Pfizer). Adenoviral vaccines (ChAdOx1, Ad26.COV2.S), however, exhibited lower antibody levels but similar T-cell responses [53]. Nevertheless, a better seroconversion response might be expected from mixed vaccination approaches [54,55]. We believe that further studies on such alternative vaccination protocols can further enhance the treatment of hematological patients.

## 6 Conclusion

In summary, despite the small cohort size, our “real world” data support the need for individualized vaccination strategies. Our findings underscore the importance of tailoring vaccination strategies, especially for patients undergoing B-cell-targeted therapies. For instance, the timing of vaccinations during periods of minimal immunosuppression or combining active and passive immunization approaches could enhance protection in these vulnerable populations. For weak-responders, alternative approaches such as passive immunization [56] may be warranted. Our data also suggest that peripheral immune status before vaccination can be a predictive biomarker for successful seroconversion. Future (meta-)studies should validate these observations using larger populations and explore the interplay between treatment timing, immune status, and vaccine efficacy in more detail and with higher statistical power.

## Supplementary Materials



Figure S1 Representative example for immune profiling using the Beckman Coulter CYTO-STAT tetraCHROME panel, 6607073 as described in Table S3. LY: lymphocytes, ADJ: CD3- cells (B- and NK-cells), ADJLN1: CD3+ T-lymphocytes, GADJ: total number of lymphocytes in CD45/sideward scatter, TADJ: total number of vital cells.

Figure S2 Seroconversion in a group of 300 healthy subjects from the second arm of this study 4 weeks after vaccination. Boxplot indicates median and quartiles of the antibody titer in these subjects.

## Data Availability

The authors confirm that the data supporting the findings of this study are available within the article and its Supplementary Materials.

## Supplementary Materials

The supplementary material is available online at https://www.techscience.com/doi/10.32604/or.2025.067561/s1.
