# Supplementary material for: SARS-CoV-2 Vaccine-Induced Seroconversion and Immune Correlates in Patients with Hematological Malignancies. A Real World Study
Source: Oncol Res. 2025 Sep 26;33(10):2923–35. doi: 10.32604/or.2025.067561 (PMC12494110; doi:10.32604/or.2025.067561)
Supplement: Supplementary file 1 [file OncolRes-33-67561-s001.docx]

**Supplementary Materials:**

Table S1: Detailed data on antibody titer and peripheral immune cells.

| Patient Nr | Date sample | time point | SARS-CoV2- Antibodies | SARS-CoV2- Antibodies | T-cells CD3 % | T-cells CD3 cells/µL | T-help CD3+ CD4+ % | T-help CD3+ CD4+ cells/µL | T-supp CD3+ CD8+ % |
| --- | --- | --- | --- | --- | --- | --- | --- | --- | --- |
| 1 | 29.01.2021 | 1 | Negative | 0 | 95.43 | 2302 | 23.75 | 573 | 74.07 |
| 2 | 29.01.2021 | 1 | 0 | 0 | 72.30 | 983 | 43.52 | 592 | 25.98 |
| 3 | 29.01.2021 | 1 | 0 | 0 | 42.02 | 367 | 21.72 | 190 | 22.64 |
| 4 | 29.01.2021 | 1 | Negative | 0 | 69.73 | 1059 | 30.75 | 467 | 38.30 |
| 5 | 29.01.2021 | 1 | Negative | 0 | 90.16 | 2268 | 10.53 | 265 | 79.80 |
| 6 | 29.01.2021 | 1 | Negative | 0 | 69.89 | 1107 | 51.07 | 809 | 18.84 |
| 7 | 29.01.2021 | 1 | negative | 0 | 33.88 | 1163 | 19.79 | 679 | 13.03 |
| 8 | 30.04.2021 | 1 | Negative | 0 | 71.51 | 1089 | 54.63 | 832 | 15.70 |
| 9 | 30.04.2021 | 1 | Negative | 0 | 63.50 | 406 | 33.03 | 211 | 27.41 |
| 10 | 30.04.2021 | 1 | Negative | 0 | 73.73 | 1785 | 57.27 | 1386 | 8.56 |
| 11 | 30.04.2021 | 1 | Negative | 0 | 56.23 | 1528 | 44.81 | 1218 | 11.91 |
| 12 | 30.04.2021 | 1 | Negative | 0 | 64.51 | 902 | 44.33 | 620 | 17.82 |
| 13 | 30.04.2021 | 1 | 68.56 | 68.56 | 65.94 | 1340 | 26.22 | 533 | 36.69 |
| 14 | 17.05.2021 | 1 | Negative | 0 | 67.77 | 1253 | 48.46 | 896 | 17.60 |
| 15 | 08.04.2021 | 1 | Negative | 0 | 64.64 | 645 | 29.30 | 292 | 30.13 |
| 16 | 08.04.2021 | 1 | Negative | 0 | 97.99 | 505 | 19.25 | 102 | 76.97 |
| 17 | 08.04.2021 | 1 | Negative | 0.00 | 81.20 | 592 | 28.29 | 206 | 56.03 |
| 18 |  | 1 | Negative | 0 |  |  |  |  |  |
| Patient Nr | T-supp CD3+ CD8+ cells/µL | B-cells CD19 % | B-cells CD19 cells/µL | Antikörper / B-cells | NK-cells CD3 - 16+ 56 + % | NK-cells CD3 - 16+ 56 + cells/µL | CD3+ HLA DR+ % | CD3+ HLA DR+ cells/µL | Ratio CD4/CD8 |
| 1 | 1787 | 0.299 | 7 | 0 | 4.14 | 100 | 14.34 | 346 | 0.32 |
| 2 | 353 | 10.36 | 141 | 0 | 17.37 | 236 | 20.71 | 282 | 1.68 |
| 3 | 198 | 37.552 | 328 | 0 | 21.99 | 192 | 20.67 | 181 | 0.96 |
| 4 | 582 | 18.721 | 284 | 0 | 11.51 | 175 | 12.26 | 186 | 0.80 |
| 5 | 2007 | 4.368 | 110 | 0 | 6.87 | 173 | 42.79 | 1076 | 0.13 |
| 6 | 298 | 4.471 | 71 | 0 | 26.33 | 471 | 12.11 | 192 | 2.71 |
| 7 | 447 | 56.101 | 1925 | 0 | 8.49 | 291 | 5.84 | 201 | 1.52 |
| 8 | 239 | 10.746 | 164 | 0 | 14.63 | 223 | 11.46 | 175 | 3.48 |
| 9 | 175 | 3.858 | 25 | 0 | 34.22 | 219 | 22.32 | 143 | 1.21 |
| 10 | 207 | 12.128 | 294 | 0 | 12.73 | 308 | 2.40 | 58 | 6.69 |
| 11 | 324 | 17.571 | 478 | 0 | 25.85 | 702 | 1.65 | 45 | 3.76 |
| 12 | 249 | 12.925 | 181 | 0 | 19.99 | 280 | 0.70 | 10 | 2.49 |
| 13 | 746 | 18.718 | 380 | 0.18 | 12.81 | 260 | 16.33 | 332 | 0.71 |
| 14 | 325 | 16.095 | 298 | 0 | 14.17 | 262 | 5.06 | 94 | 2.75 |
| 15 | 301 | 19.579 | 195 | 0 | 16.47 | 164 | 17.16 | 171 | 0.97 |
| 16 | 409 | 0.551 | 3.1 | 0 | 1.65 | 9 | 79.68 | 423 | 0.25 |
| 17 | 408 | 3.855 | 28 | 0 | 12.03 | 88 | 22.80 | 167 | 0.51 |
| Patient Nr | Date sample | time point | SARS-CoV2- Antibodies | SARS-CoV2- Antibodies | T-cells CD3 % | T-cells CD3 cells/µL | T-help CD3+ CD4+ % | T-help CD3+ CD4+ cells/µL | T-supp CD3+ CD8+ % |
| 1 | 18.02.2021 | 2 | Negative | 0 | 94.81 | 3441 | 21.34 | 775 | 74.41 |
| 2 | 18.02.2021 | 2 | 0 | 0 | 67.25 | 943 | 41.14 | 577 | 25.81 |
| 3 | 18.02.2021 | 2 | 30.8 | 30.8 | 44.44 | 493 | 19.05 | 211 | 26.54 |
| 4 | 18.02.2021 | 2 | Negative | 0 | 66.83 | 776 | 33.20 | 386 | 34.32 |
| 5 | 18.02.2021 | 2 | Negative | 0 | 87.12 | 1340 | 11.39 | 175 | 76.17 |
| 6 | 18.02.2021 | 2 | Negative | 0 | 63.35 | 851 | 45.38 | 610 | 17.44 |
| 7 | 18.02.2021 | 2 | negative | 0 | 37.71 | 1572 | 21.15 | 882 | 15.50 |
| 9 | 17.05.2021 | 2 |  |  |  |  |  |  |  |
| 10 | 17.05.2021 | 2 | Negative | 0 | 76.37 | 1626 | 60.12 | 1280 | 9.31 |
| 11 | 17.05.2021 | 2 | Negative | 0 | 59.12 | 1216 | 46.65 | 960 | 13.54 |
| 13 | 17.05.2021 | 2 | >2500 | 2500 | 62.95 | 823 | 27.39 | 358 | 34.92 |
| 14 | 11.06.2021 | 2 | Negative | 0 | 71.87 | 1379 | 44.00 | 845 | 22.58 |
| 15 | 28.04.2021 | 2 | Negative | 0 | 61.71 | 924 | 26.56 | 398 | 28.00 |
| 16 | 28.04.2021 | 2 | Negative | 0 | 97.77 | 595 | 18.19 | 111 | 79.86 |
| 17 | 28.04.2021 | 2 | Negative | 0 | 79.43 | 697 | 28.17 | 247 | 56.78 |
| 18 | 16.04.2021 | 2 | Negative | 0 |  |  |  |  |  |
| Patient Nr | T-supp CD3+ CD8+ cells/µL | B-cells CD19 % | B-cells CD19 cells/µL | Antikörper / B-cells | NK-cells CD3 - 16+ 56 + % | NK-cells CD3 - 16+ 56 + cells/µL | CD3+ HLA DR+ % | CD3+ HLA DR+ cells/µL | Ratio CD4/CD8 |
| 1 | 2701 | 0.288 | 10 | 0 | 4.51 | 164 | 18.62 | 676 | 0.29 |
| 2 | 362 | 12.783 | 179 | 0 | 20.23 | 284 | 22.08 | 310 | 1.59 |
| 3 | 294 | 34.534 | 383 | 0.08 | 20.66 | 229 | 23.77 | 264 | 0.72 |
| 4 | 398 | 23.873 | 277 | 0 | 10.08 | 117 | 14.82 | 172 | 0.97 |
| 5 | 1172 | 6.955 | 107 | 0 | 8.25 | 127 | 48.14 | 740 | 0.15 |
| 6 | 234 | 4.941 | 66 | 0 | 31.07 | 418 | 14.36 | 193 | 2.60 |
| 7 | 646 | 51.371 | 2142 | 0 | 9.10 | 379 | 7.97 | 332 | 1.36 |
| 8 | 263 | 16.869 | 266 | 0 | 10.43 | 164 | 15.56 | 245 | 3.21 |
| 10 | 198 | 12.146 | 259 | 0 | 11.42 | 243 | 2.71 | 58 | 6.46 |
| 11 | 278 | 16.938 | 348 | 0 | 24.68 | 508 | 2.11 | 43 | 3.45 |
| 13 | 456 | 14.004 | 183 | 13.67 | 21.85 | 285 | 25.64 | 335 | 0.78 |
| 14 | 433 | 12.516 | 240 | 0 | 15.94 | 306 | 9.37 | 180 | 1.95 |
| 15 | 419 | 18.083 | 271 | 0 | 20.18 | 302 | 18.47 | 277 | 0.95 |
| 16 | 486 | 0.669 | 4 |  | 1.51 | 9 | 87.78 | 535 | 0.23 |
| 17 | 498 | 4.123 | 36 |  | 12.32 | 108 | 25.26 | 222 | 0.50 |
| Patient Nr | Date sample | time point | SARS-CoV2- Antibodies | SARS-CoV2- Antibodies | T-cells CD3 % | T-cells CD3 cells/µL | T-help CD3+ CD4+ % | T-help CD3+ CD4+ cells/µL | T-supp CD3+ CD8+ % |
| 1 | 01.03.- 07.03. | 3 | Negative | 0 | 94.36 | 3916 | 22.57 | 936 | 73.21 |
| 2 | 01.03.- 07.03. | 3 | 313.3 | 313.3 | 67.85 | 821 | 46.00 | 557 | 21.91 |
| 3 | 01.03.- 07.03. | 3 | 2287 | 2287 | 53.26 | 692 | 15.80 | 205 | 37.97 |
| 4 | 01.03.- 07.03. | 3 | 456.3 | 456.3 | 69.38 | 1094 | 34.08 | 538 | 35.47 |
| 5 | 01.03.- 07.03. | 3 | 124.4 | 124.4 | 85.31 | 1641 | 10.73 | 206 | 74.62 |
| 6 | 01.03.- 07.03. | 3 | 23.8 | 23.8 | 63.34 | 995 | 45.74 | 718 | 18.36 |
| 7 | 01.03.- 07.03. | 3 | negative | 0 | 36.08 | 1591 | 20.89 | 921 | 14.36 |
| 8 | 11.06.201 | 3 | Negative | 0 |  |  |  |  |  |
| 10 | 07.06.2021 | 3 | Negative | 0 |  |  |  |  |  |
| 11 | 11.06.2021 | 3 | Negative | 0 |  |  |  |  |  |
| 14 | 12.07.2021 | 3 | 24.22 | 24.22 |  |  |  |  |  |
| 15 | 17.05-23.05 | 3 | >2500 | 2500 | 64.24 | 1055 | 25.38 | 417 | 31.93 |
| 16 | 17.05-23.05 | 3 | Negative | 0 | 97.64 | 555 | 18.05 | 103 | 78.71 |
| 17 | 17.05-23.05 | 3 | Negative | 0 |  |  |  |  |  |
| Patient Nr | T-supp CD3+ CD8+ cells/µL | B-cells CD19 % | B-cells CD19 cells/µL | Antikörper / B-cells | NK-cells CD3 - 16+ 56 + % | NK-cells CD3 - 16+ 56 + cells/µL | CD3+ HLA DR+ % | CD3+ HLA DR+ cells/µL | Ratio CD4/CD8 |
| 1 | 3038 | 0.285 | 12 | 0 | 4.84 | 201 | 14.36 | 596 | 0.31 |
| 2 | 265 | 16.845 | 204 | 1.53 | 14.23 | 172 | 15.38 | 186 | 2.10 |
| 3 | 493 | 31.394 | 408 | 5.60 | 14.77 | 192 | 34.45 | 448 | 0.42 |
| 4 | 560 | 20.89 | 330 | 1.38 | 10.23 | 161 | 17.10 | 270 | 0.96 |
| 5 | 1435 | 9.319 | 179 | 0.69 | 8.56 | 165 | 37.80 | 727 | 0.14 |
| 6 | 288 | 3.994 | 63 | 0.37 | 29.17 | 458 | 11.05 | 173 | 2.49 |
| 7 | 633 | 54.906 | 2421 | 0 | 7.89 | 348 | 5.95 | 262 | 1.46 |
| 15 | 524 | 17.203 | 282 | 8.86 | 18.38 | 302 | 18.83 | 309 | 0.80 |
| 16 | 447 | 0.542 | 3 |  | 1.75 | 10 | 83.61 | 475 | 0.23 |
| Patient Nr | Date sample | time point | SARS-CoV2- Antibodies | SARS-CoV2- Antibodies | T-cells CD3 % | T-cells CD3 cells/µL | T-help CD3+ CD4+ % | T-help CD3+ CD4+ cells/µL | T-supp CD3+ CD8+ % |
| 1 | 15.03.-21.03 | 4 | Negative | 0 |  |  |  |  |  |
| 2 | 15.03.-21.03 | 4 | 164.6 | 164.6 |  |  |  |  |  |
| 3 | 15.03.-21.03 | 4 | 1516 | 1516 |  |  |  |  |  |
| 4 | 15.03.-21.03 | 4 | 819.4 | 819.4 |  |  |  |  |  |
| 5 | 15.03.-21.03 | 4 | 105.8 | 105.8 |  |  |  |  |  |
| 7 | 15.03.-21.03 | 4 | 14.29 | 14.29 |  |  |  |  |  |
| 8 | 25.06.2021 | 4 | >2500 | 2500 |  |  |  |  |  |
| 10 | 25.06.2021 | 4 | >2500 | 2500 |  |  |  |  |  |
| 11 | 25.06.2021 | 4 | 757 | 757 |  |  |  |  |  |
| 13 | 12.07.2021 | 4 | >2500 | 2500 |  |  |  |  |  |
| 14 | 14.09.2021 | 4 | >2500 | 2500 |  |  |  |  |  |
| 15 | 08.06.2021 | 4 | 1481 | 1481 |  |  |  |  |  |
| 16 | 26.05.2021 | 4 | Negative | 0 |  |  |  |  |  |
| 17 | 07.06.2021 | 4 | Negative | 0 |  |  |  |  |  |
| Patient Nr | Date sample | time point | SARS-CoV2- Antibodies | SARS-CoV2- Antibodies | T-cells CD3 % | T-cells CD3 cells/µL | T-help CD3+ CD4+ % | T-help CD3+ CD4+ cells/µL | T-supp CD3+ CD8+ % |
| 1 | 15.04.-22.04 | 5 | Negative | 0 | 94.81 | 3507 | 22.58 | 835 | 72.80 |
| 2 | 15.04.-22.04 | 5 | 187.4 | 187.4 | 65.41 | 1053 | 45.23 | 728 | 21.79 |
| 3 | 15.04.-22.04 | 5 | 1131 | 1131 | 50.50 | 691 | 15.56 | 213 | 36.52 |
| 4 | 15.04.-22.04 | 5 | 514.8 | 514.8 | 60.22 | 1023 | 29.55 | 502 | 30.48 |
| 5 | 15.04.-22.04 | 5 | 131.6 | 131.6 | 83.33 | 1066 | 15.31 | 196 | 68.55 |
| 6 | 15.04.-22.04 | 5 | 6.77 | 6.77 | 83.05 | 745 | 58.48 | 524 | 24.35 |
| 7 | 15.04.-22.04 | 5 | 85.3 | 85.3 | 34.19 | 1500 | 20.37 | 893 | 12.96 |
| 8 | 12.07.2021 | 5 | 2066 | 2066 |  |  |  |  |  |
| 10 | 13.07.2021 | 5 | >2500 | 2500 |  |  |  |  |  |
| 11 | 11.08.2021 | 5 | 1382 | 1382 |  |  |  |  |  |
| 14 | 11.10.2021 | 5 |  |  | 72.38 | 1375 | 46.51 | 884 | 21.69 |
| 15 | 05.07.2021 | 5 | 562.7 | 562.7 | 85.08 | 734 | 33.16 | 286 | 48.04 |
| 16 | 30.06.2021 | 5 | 9.8 | 9.8 | 96.48 | 926 | 17.37 | 167 | 76.29 |
| 17 | 06.07.2021 | 5 | Negative | 0 |  |  |  |  |  |
| 18 | 09.09.2021 | 5 | >2500 | 2500 |  |  |  |  |  |
| Patient Nr | T-supp CD3+ CD8+ cells/µL | B-cells CD19 % | B-cells CD19 cells/µL | Antikörper / B-cells | NK-cells CD3 - 16+ 56 + % | NK-cells CD3 - 16+ 56 + cells/µL | CD3+ HLA DR+ % | CD3+ HLA DR+ cells/µL | Ratio CD4/CD8 |
| 1 | 2693 | 0.539 | 20 | 0 | 4.12 | 152 | 12.48 | 462 | 0.31 |
| 2 | 351 | 17.229 | 277 | 0.67 | 15.96 | 257 | 11.89 | 191 | 2.08 |
| 3 | 500 | 29.85 | 408 | 2.77 | 15.52 | 212 | 23.78 | 325 | 0.43 |
| 4 | 518 | 30.106 | 511 | 1.01 | 9.55 | 162 | 12.79 | 217 | 0.97 |
| 5 | 877 | 9.518 | 122 | 1.08 | 6.76 | 86 | 23.59 | 302 | 0.22 |
| 6 | 218 | 0.024 | 0 |  | 14.03 | 126 | 14.96 | 134 | 2.40 |
| 7 | 568 | 56.398 | 2474 | 0.03 | 8.31 | 364 | 4.16 | 182 | 1.57 |
| 14 | 412 | 16.236 | 309 | 0 | 11.07 | 210 | 9.67 | 184 | 2.15 |
| 15 | 415 | 11.557 | 100 | 5.63 | 2.88 | 25 | 54.20 | 468 | 0.69 |
| 16 | 732 | 1.997 | 19 |  | 2.00 | 19 | 60.18 | 578 | 0.23 |
| Patient Nr | Date sample | time point | SARS-CoV2- Antibodies | SARS-CoV2- Antibodies | T-cells CD3 % | T-cells CD3 cells/µL | T-help CD3+ CD4+ % | T-help CD3+ CD4+ cells/µL | T-supp CD3+ CD8+ % |
| 1 | 13.05.-20.05. | 6 | Negative | 0 | 95.21 | 3272 | 22.81 | 784 | 72.92 |
| 2 | 13.05.-20.05. | 6 | 155.4 | 155.4 | 64.38 | 1374 | 43.84 | 936 | 23.13 |
| 3 | 13.05.-20.05. | 6 | 1065 | 1065 | 55.42 | 781 | 20.30 | 286 | 36.06 |
| 4 | 13.05.-20.05. | 6 | 315.3 | 315.3 | 55.50 | 1188 | 27.46 | 588 | 30.24 |
| 5 | 13.05.-20.05. | 6 | 51.44 | 51.44 | 96.16 | 2625 | 8.00 | 219 | 89.35 |
| 6 | 13.05.-20.05. | 6 | Negative | 0 | 94.92 | 1905 | 73.77 | 1480 | 22.61 |
| 7 | 13.05.-20.05. | 6 | 73.83 | 73.83 | 21.91 | 877 | 12.21 | 489 | 8.71 |
| 8 | 13.09.2021 | 6 | 906 | 906 | 63.83 | 773 | 52.71 | 638 | 11.43 |
| 9 | 13.05.-20.05. | 6 |  |  |  |  |  |  |  |
| 10 | 11.08.2021 | 6 | >2500 | 2500 |  |  |  |  |  |
| 13 | 11.10.2021 | 6 | >2500 | 2500 | 56.21 | 546 | 2.17 | 21 | 0.89 |
| 14 | 17.06.-24.06. | 5 | 974 | 974 |  |  |  |  |  |
| 15 | 09.08.2021 | 6 | 255 | 255 |  |  |  |  |  |
| 16 | 30.08.2021 | 6 | 35.95 | 35.95 |  |  |  |  |  |
| 17 | 09.08.2021 | 6 | Negative | 0 |  |  |  |  |  |
| Patient Nr | T-supp CD3+ CD8+ cells/µL | B-cells CD19 % | B-cells CD19 cells/µL | Antikörper / B-cells | NK-cells CD3 - 16+ 56 + % | NK-cells CD3 - 16+ 56 + cells/µL | CD3+ HLA DR+ % | CD3+ HLA DR+ cells/µL | Ratio CD4/CD8 |
| 1 | 2507 | 0.435 | 15 | 0 | 4.11 | 141 | 11.83 | 407 | 0.31 |
| 2 | 494 | 18.244 | 389 | 0.40 | 17.31 | 369 | 12.00 | 256 | 1.90 |
| 3 | 508 | 29.369 | 414 | 2.57 | 15.43 | 217 | 28.89 | 407 | 0.56 |
| 4 | 648 | 38.014 | 814 | 0.39 | 8.25 | 177 | 12.29 | 263 | 0.91 |
| 5 | 2439 | 0.171 | 5 | 10.29 | 3.44 | 94 | 78.40 | 2140 | 0.09 |
| 6 | 454 | 0.089 | 2 | 0 | 4.25 | 85 | 7.80 | 157 | 3.26 |
| 7 | 349 | 72.511 | 2903 | 0.03 | 4.79 | 192 | 3.21 | 129 | 1.40 |
| 8 | 138 | 24.681 | 299 | 3.03 | 11.43 | 138 | 5.79 | 70 | 4.61 |
| 13 | 9 | 26.991 | 262 | 9.54 | 0.75 | 7 | 0.63 | 6 |  |
| Patient Nr | Date sample | time point | SARS-CoV2- Antibodies | SARS-CoV2- Antibodies | T-cells CD3 % | T-cells CD3 cells/µL | T-help CD3+ CD4+ % | T-help CD3+ CD4+ cells/µL | T-supp CD3+ CD8+ % |
| 1 | 17.06.-24.06. | 7 | Negative | 0 |  |  |  |  |  |
| 2 | 17.06.-24.06. | 7 | 163.3 | 163.3 |  |  |  |  |  |
| 3 | 17.06.-24.06. | 7 | 974 | 974 |  |  |  |  |  |
| 4 | 17.06.-24.06. | 7 | 204 | 204 |  |  |  |  |  |
| 5 | 17.06.-24.06. | 7 | 22.91 | 22.91 |  |  |  |  |  |
| 6 | 17.06.-24.06. | 7 | Negative | 0 |  |  |  |  |  |
| 7 | 17.06.-24.06. | 7 | 73.56 | 73.56 |  |  |  |  |  |
| 10 | 14.09.2021 | 7 |  |  | 76.27 | 1493 | 62.49 | 1224 | 8.98 |
| 13 |  | 7 | >2500 | 2500 |  |  |  |  |  |
| 14 | 15.10.-22.10 | 6 |  |  |  |  |  |  |  |
| 15 | 10.09.2021 | 5 | 152 | 152 | 85.08 | 734 | 33.16 | 286 | 48.04 |
| Patient Nr | T-supp CD3+ CD8+ cells/µL | B-cells CD19 % | B-cells CD19 cells/µL | Antikörper / B-cells | NK-cells CD3 - 16+ 56 + % | NK-cells CD3 - 16+ 56 + cells/µL | CD3+ HLA DR+ % | CD3+ HLA DR+ cells/µL | Ratio CD4/CD8 |
| 10 | 176 | 12.335 | 242 | 0 | 11.84 | 232 | 1.60 | 31 | 6.96 |
| 15 | 415 | 11.557 | 100 | 1.52 | 2.88 | 25 | 54.20 | 468 | 0.69 |
| Patient Nr | Date sample | time point | SARS-CoV2- Antibodies | SARS-CoV2- Antibodies | T-cells CD3 % | T-cells CD3 cells/µL | T-help CD3+ CD4+ % | T-help CD3+ CD4+ cells/µL | T-supp CD3+ CD8+ % |
| 1 | 06.10.2021 | 8 | 10.3 | 10.3 | 94.75 | 2482 | 23.65 | 620 | 72.09 |
| 2 | 15.10.-22.10 | 8 | 109 | 109 | 53.75 | 1123 | 34.00 | 710 | 21.45 |
| 3 | 15.10.-22.10 | 8 | 726 | 726 | 51.29 | 809 | 26.19 | 413 | 24.30 |
| 4 | 15.10.-22.10 | 8 | 126 | 126 | 34.96 | 1547 | 17.91 | 792 | 17.74 |
| 5 | 01.10.2021 | 8 | Negative | 0 | 83.66 | 1747 | 11.38 | 238 | 73.40 |
| 6 | 15.10.-22.10 | 8 | Negative | 0 | 82.08 | 948 | 60.87 | 730 | 23.30 |
| 7 | 15.10.-22.10 | 8 | 64.1 | 64.1 | 93.16 | 1005 | 64.87 | 700 | 27.13 |
| Patient Nr | T-supp CD3+ CD8+ cells/µL | B-cells CD19 % | B-cells CD19 cells/µL | Antikörper / B-cells | NK-cells CD3 - 16+ 56 + % | NK-cells CD3 - 16+ 56 + cells/µL | CD3+ HLA DR+ % | CD3+ HLA DR+ cells/µL | Ratio CD4/CD8 |
| 1 | 1888 | 0.179 | 5 | 2.06 | 4.49 | 118 | 14.33 | 375 | 0.33 |
| 2 | 448 | 16.54 | 345 | 0.32 | 24.73 | 517 | 8.71 | 182 | 1.59 |
| 3 | 384 | 35.481 | 560 | 1.29 | 11.52 | 182 | 13.44 | 212 | 1.08 |
| 4 | 785 | 62.144 | 2749 | 0.05 | 3.02 | 1347 | 5.45 | 241 | 1.01 |
| 5 | 1533 | 11.032 | 230 | 0 | 8.58 | 179 | 25.47 | 532 | 0.16 |
| 6 | 279 | 1.449 | 17 | 0 | 15.98 | 192 | 12.50 | 150 | 2.62 |
| 7 | 293 | 0.132 | 1 | 64.1 | 5.85 | 63 | 15.33 | 165 | 2.39 |

Table S2:

Descriptive statistics for peripheral antibodies and immune cells according to weak (0) and good (1) responders.

| **Descriptive Statistics** | | | | | | |  |
| --- | --- | --- | --- | --- | --- | --- | --- |
|  | | N | Average | Standard Deviation | 95% CI | | **ANOVA** |
|  |  |  |  |  | Lower | Upper | Sig. |
| SARS-CoV2- Antibodies  time point 1 | 0 | 7.000 | 0.000 | 0.000 | 0.000 | 0.000 | 0.369 |
|  | 1 | 8.000 | 8.570 | 24.240 | -11.695 | 28.835 |  |
|  | all | 15.000 | 4.571 | 17.702 | -5.232 | 14.374 |  |
| SARS-CoV2- Antibodies  time point 2 | 0 | 7.000 | 0.000 | 0.000 | 0.000 | 0.000 | 0.362 |
|  | 1 | 8.000 | 316.350 | 882.394 | -421.350 | 1054.050 |  |
|  | all | 15.000 | 168.720 | 644.978 | -188.457 | 525.897 |  |
| SARS-CoV2- Antibodies  time point 3 | 0 | 7.000 | 65.929 | 118.161 | -43.352 | 175.210 | 0.132 |
|  | 1 | 7.000 | 760.287 | 1128.855 | -283.729 | 1804.304 |  |
|  | all | 14.000 | 413.108 | 851.113 | -78.310 | 904.526 |  |
| SARS-CoV2- Antibodies  time point 4 | 0 | 6.000 | 47.448 | 70.687 | -26.733 | 121.629 | < 0.001 |
|  | 1 | 8.000 | 1821.675 | 773.497 | 1175.015 | 2468.335 |  |
|  | all | 14.000 | 1061.292 | 1074.380 | 440.964 | 1681.621 |  |
| SARS-CoV2- Antibodies  time point 5 | 0 | 7.000 | 60.124 | 75.885 | -10.057 | 130.306 | 0.001 |
|  | 1 | 6.000 | 1359.417 | 800.126 | 519.736 | 2199.098 |  |
|  | all | 13.000 | 659.798 | 850.961 | 145.567 | 1174.028 |  |
| SARS-CoV2- Antibodies  time point 6 | 0 | 7.000 | 45.231 | 56.594 | -7.109 | 97.572 | 0.009 |
|  | 1 | 6.000 | 1002.550 | 810.868 | 151.596 | 1853.504 |  |
|  | all | 13.000 | 487.071 | 722.704 | 50.345 | 923.797 |  |
| SARS-CoV2- Antibodies  time point 7 | 0 | 5.000 | 51.954 | 69.114 | -33.862 | 137.770 | 0.102 |
|  | 1 | 4.000 | 957.500 | 1094.862 | -784.670 | 2699.670 |  |
|  | all | 9.000 | 454.419 | 824.434 | -179.297 | 1088.135 |  |
| SARS-CoV2- Antibodies  time point 8 | 0 | 5.000 | 36.680 | 48.396 | -23.412 | 96.772 | 0.062 |
|  | 1 | 2.000 | 426.000 | 424.264 | -3385.861 | 4237.861 |  |
|  | all | 7.000 | 147.914 | 260.095 | -92.634 | 388.462 |  |
|  |  |  |  |  |  |  |  |
| **descriptive statistic** | | | | | | |  |
|  | | N | Average | Standard deviation | 95% CI | | **ANOVA** |
|  |  |  |  |  | lower | upper | Sig. |
| T-cells CD3 %  time point 1 | 0 | 7.000 | 77.265 | 21.997 | 56.921 | 97.608 | 0.135 |
|  | 1 | 7.000 | 62.548 | 10.299 | 53.023 | 72.073 |  |
|  | all | 14.000 | 69.906 | 18.182 | 59.408 | 80.404 |  |
| T-cells CD3 cells/µL  time point 1 | 0 | 7.000 | 1274.286 | 733.280 | 596.115 | 1952.456 | 0.474 |
|  | 1 | 7.000 | 1040.143 | 405.222 | 665.375 | 1414.911 |  |
|  | all | 14.000 | 1157.214 | 581.993 | 821.182 | 1493.247 |  |
| T-help CD3+ CD4+ %  time point 1 | 0 | 7.000 | 28.029 | 14.378 | 14.732 | 41.326 | 0.261 |
|  | 1 | 7.000 | 36.555 | 12.584 | 24.917 | 48.194 |  |
|  | all | 14.000 | 32.292 | 13.714 | 24.374 | 40.211 |  |
| T-help CD3+ CD4+ cells/µL  time point 1 | 0 | 7.000 | 460.857 | 267.910 | 213.082 | 708.633 | 0.336 |
|  | 1 | 7.000 | 632.571 | 365.395 | 294.638 | 970.505 |  |
|  | all | 14.000 | 546.714 | 320.449 | 361.693 | 731.736 |  |
| T-supp CD3+ CD8+ %  time point 1 | 0 | 7.000 | 49.246 | 29.274 | 22.171 | 76.320 | 0.059 |
|  | 1 | 7.000 | 24.710 | 10.469 | 15.028 | 34.392 |  |
|  | all | 14.000 | 36.978 | 24.662 | 22.739 | 51.217 |  |
| T-supp CD3+ CD8+ cells/µL  time point 1 | 0 | 7.000 | 815.571 | 742.999 | 128.412 | 1502.731 | 0.167 |
|  | 1 | 7.000 | 387.857 | 199.889 | 202.990 | 572.724 |  |
|  | all | 14.000 | 601.714 | 567.878 | 273.831 | 929.597 |  |
| B-cells CD19 %  time point 1 | 0 | 7.000 | 11.429 | 19.977 | -7.047 | 29.905 | 0.324 |
|  | 1 | 7.000 | 19.855 | 8.348 | 12.134 | 27.575 |  |
|  | all | 14.000 | 15.642 | 15.345 | 6.782 | 24.502 |  |
| B-cells CD19 cells/µL  time point 1 | 0 | 7.000 | 326.443 | 706.816 | -327.253 | 980.139 | 0.935 |
|  | 1 | 7.000 | 303.857 | 106.884 | 205.006 | 402.708 |  |
|  | all | 14.000 | 315.150 | 485.788 | 34.665 | 595.635 |  |
| Antikörper / B-cells  time point 1 | 0 | 7.000 | 0.000 | 0.000 | 0.000 | 0.000 | 0.369 |
|  | 1 | 8.000 | 0.023 | 0.064 | -0.031 | 0.076 |  |
|  | all | 15.000 | 0.012 | 0.047 | -0.014 | 0.038 |  |
| NK-cells CD3 - 16+ 56 + %  time point 1 | 0 | 7.000 | 10.983 | 8.509 | 3.113 | 18.852 | 0.151 |
|  | 1 | 7.000 | 16.775 | 5.233 | 11.936 | 21.615 |  |
|  | all | 14.000 | 13.879 | 7.422 | 9.593 | 18.164 |  |
| NK-cells CD3 - 16+ 56 + cells/µL  time point 1 | 0 | 7.000 | 195.429 | 154.178 | 52.837 | 338.020 | 0.363 |
|  | 1 | 7.000 | 282.571 | 188.960 | 107.812 | 457.330 |  |
|  | all | 14.000 | 239.000 | 171.742 | 139.839 | 338.161 |  |
| CD3+ HLA DR+ %  time point 1 | 0 | 7.000 | 28.326 | 25.491 | 4.751 | 51.901 | 0.129 |
|  | 1 | 7.000 | 12.084 | 6.782 | 5.811 | 18.356 |  |
|  | all | 14.000 | 20.205 | 19.803 | 8.771 | 31.639 |  |
| CD3+ HLA DR+ cells/µL  time point 1 | 0 | 7.000 | 383.857 | 318.767 | 89.047 | 678.668 | 0.112 |
|  | 1 | 7.000 | 169.143 | 89.483 | 86.385 | 251.900 |  |
|  | all | 14.000 | 276.500 | 251.010 | 131.571 | 421.429 |  |
| Ratio CD4/CD8  time point 1 | 0 | 7.000 | 1.016 | 0.972 | 0.117 | 1.915 | 0.177 |
|  | 1 | 7.000 | 1.921 | 1.357 | 0.666 | 3.175 |  |
|  | all | 14.000 | 1.468 | 1.227 | 0.760 | 2.177 |  |
|  |  |  |  |  |  |  |  |
| SARS-CoV2- Antibodies  time point 2 | 0 | 7.000 | 0.000 | 0.000 | 0.000 | 0.000 | 0.362 |
|  | 1 | 8.000 | 316.350 | 882.394 | -421.350 | 1054.050 |  |
|  | all | 15.000 | 168.720 | 644.978 | -188.457 | 525.897 |  |
| T-cells CD3 %  time point 2 | 0 | 7.000 | 75.345 | 21.067 | 55.861 | 94.829 | 0.173 |
|  | 1 | 7.000 | 62.708 | 9.449 | 53.969 | 71.446 |  |
|  | all | 14.000 | 69.026 | 17.001 | 59.210 | 78.842 |  |
| T-cells CD3 cells/µL  time point 2 | 0 | 7.000 | 1348.429 | 985.962 | 436.565 | 2260.292 | 0.343 |
|  | 1 | 7.000 | 963.714 | 301.008 | 685.328 | 1242.100 |  |
|  | all | 14.000 | 1156.071 | 728.243 | 735.597 | 1576.546 |  |
| T-help CD3+ CD4+ %  time point 2 | 0 | 7.000 | 26.680 | 12.423 | 15.190 | 38.169 | 0.198 |
|  | 1 | 7.000 | 35.779 | 12.553 | 24.169 | 47.389 |  |
|  | all | 14.000 | 31.229 | 12.894 | 23.785 | 38.674 |  |
| T-help CD3+ CD4+ cells/µL  time point 2 | 0 | 7.000 | 482.429 | 305.061 | 200.295 | 764.563 | 0.591 |
|  | 1 | 7.000 | 571.714 | 300.013 | 294.249 | 849.180 |  |
|  | all | 14.000 | 527.071 | 294.347 | 357.121 | 697.022 |  |
| T-supp CD3+ CD8+ %  time point 2 | 0 | 7.000 | 49.423 | 29.014 | 22.590 | 76.257 | 0.055 |
|  | 1 | 7.000 | 25.228 | 8.193 | 17.651 | 32.804 |  |
|  | all | 14.000 | 37.326 | 24.024 | 23.455 | 51.196 |  |
| T-supp CD3+ CD8+ cells/µL  time point 2 | 0 | 7.000 | 871.286 | 860.458 | 75.495 | 1667.077 | 0.146 |
|  | 1 | 7.000 | 363.000 | 81.543 | 287.585 | 438.415 |  |
|  | all | 14.000 | 617.143 | 643.695 | 245.484 | 988.801 |  |
| B-cells CD19 %  time point 2 | 0 | 7.000 | 11.590 | 18.039 | -5.093 | 28.273 | 0.303 |
|  | 1 | 7.000 | 19.545 | 7.523 | 12.587 | 26.503 |  |
|  | all | 14.000 | 15.568 | 13.905 | 7.539 | 23.596 |  |
| B-cells CD19 cells/µL  time point 2 | 0 | 7.000 | 363.429 | 786.645 | -364.096 | 1090.954 | 0.787 |
|  | 1 | 7.000 | 281.143 | 66.482 | 219.658 | 342.628 |  |
|  | all | 14.000 | 322.286 | 538.022 | 11.641 | 632.930 |  |
| NK-cells CD3 - 16+ 56 + %  time point 2 | 0 | 7.000 | 12.428 | 10.155 | 3.036 | 21.819 | 0.255 |
|  | 1 | 7.000 | 17.687 | 5.699 | 12.417 | 22.958 |  |
|  | all | 14.000 | 15.058 | 8.369 | 10.226 | 19.889 |  |
| NK-cells CD3 - 16+ 56 + cells/µL  time point 2 | 0 | 7.000 | 212.714 | 151.140 | 72.933 | 352.495 | 0.434 |
|  | 1 | 7.000 | 273.000 | 126.130 | 156.350 | 389.650 |  |
|  | all | 14.000 | 242.857 | 137.346 | 163.556 | 322.158 |  |
| CD3+ HLA DR+ %  time point 2 | 0 | 7.000 | 32.030 | 27.638 | 6.470 | 57.591 | 0.159 |
|  | 1 | 7.000 | 15.677 | 8.136 | 8.152 | 23.202 |  |
|  | all | 14.000 | 23.854 | 21.333 | 11.536 | 36.171 |  |
| CD3+ HLA DR+ cells/µL  time point 2 | 0 | 7.000 | 429.714 | 220.256 | 226.011 | 633.418 | 0.037 |
|  | 1 | 7.000 | 216.571 | 95.031 | 128.682 | 304.461 |  |
|  | all | 14.000 | 323.143 | 196.951 | 209.427 | 436.859 |  |
| Ratio CD4/CD8  time point 2 | 0 | 7.000 | 0.960 | 0.924 | 0.106 | 1.815 | 0.205 |
|  | 1 | 7.000 | 1.718 | 1.176 | 0.630 | 2.806 |  |
|  | all | 14.000 | 1.339 | 1.089 | 0.710 | 1.968 |  |
|  |  |  |  |  |  |  |  |
| T-cells CD3 %  time point 3 | 0 | 6.000 | 74.097 | 23.197 | 49.754 | 98.441 | 0.433 |
|  | 1 | 3.000 | 62.293 | 8.237 | 41.832 | 82.753 |  |
|  | all | 9.000 | 70.162 | 19.700 | 55.019 | 85.305 |  |
| T-cells CD3 cells/µL  time point 3 | 0 | 6.000 | 1586.500 | 1218.968 | 307.271 | 2865.729 | 0.412 |
|  | 1 | 3.000 | 947.000 | 221.696 | 396.277 | 1497.723 |  |
|  | all | 9.000 | 1373.333 | 1021.374 | 588.236 | 2158.431 |  |
| T-help CD3+ CD4+ %  time point 3 | 0 | 6.000 | 27.330 | 14.924 | 11.669 | 42.991 | 0.821 |
|  | 1 | 3.000 | 25.086 | 9.145 | 2.369 | 47.802 |  |
|  | all | 9.000 | 26.582 | 12.703 | 16.818 | 36.346 |  |
| T-help CD3+ CD4+ cells/µL  time point 3 | 0 | 6.000 | 573.500 | 354.892 | 201.064 | 945.936 | 0.427 |
|  | 1 | 3.000 | 386.667 | 168.560 | -32.059 | 805.392 |  |
|  | all | 9.000 | 511.222 | 307.486 | 274.868 | 747.577 |  |
| T-supp CD3+ CD8+ %  time point 3 | 0 | 6.000 | 46.860 | 31.529 | 13.772 | 79.948 | 0.554 |
|  | 1 | 3.000 | 35.125 | 3.035 | 27.585 | 42.664 |  |
|  | all | 9.000 | 42.948 | 25.652 | 23.230 | 62.666 |  |
| T-supp CD3+ CD8+ cells/µL  time point 3 | 0 | 6.000 | 1017.667 | 1079.672 | -115.380 | 2150.713 | 0.471 |
|  | 1 | 3.000 | 525.667 | 33.531 | 442.371 | 608.962 |  |
|  | all | 9.000 | 853.667 | 888.457 | 170.739 | 1536.595 |  |
| B-cells CD19 %  time point 3 | 0 | 6.000 | 14.315 | 20.843 | -7.558 | 36.188 | 0.511 |
|  | 1 | 3.000 | 23.162 | 7.363 | 4.871 | 41.454 |  |
|  | all | 9.000 | 17.264 | 17.454 | 3.848 | 30.680 |  |
| B-cells CD19 cells/µL  time point 3 | 0 | 6.000 | 480.333 | 954.432 | -521.281 | 1481.948 | 0.813 |
|  | 1 | 3.000 | 340.000 | 63.592 | 182.028 | 497.972 |  |
|  | all | 9.000 | 433.556 | 758.467 | -149.454 | 1016.565 |  |
| NK-cells CD3 - 16+ 56 + %  time point 3 | 0 | 6.000 | 11.071 | 9.795 | 0.792 | 21.350 | 0.593 |
|  | 1 | 3.000 | 14.462 | 4.081 | 4.323 | 24.600 |  |
|  | all | 9.000 | 12.201 | 8.185 | 5.909 | 18.493 |  |
| NK-cells CD3 - 16+ 56 + cells/µL  time point 3 | 0 | 6.000 | 225.667 | 156.605 | 61.320 | 390.013 | 0.942 |
|  | 1 | 3.000 | 218.333 | 74.097 | 34.267 | 402.400 |  |
|  | all | 9.000 | 223.222 | 129.283 | 123.846 | 322.598 |  |
| CD3+ HLA DR+ %  time point 3 | 0 | 6.000 | 28.024 | 29.348 | -2.776 | 58.823 | 0.806 |
|  | 1 | 3.000 | 23.462 | 9.556 | -0.276 | 47.200 |  |
|  | all | 9.000 | 26.503 | 23.798 | 8.210 | 44.796 |  |
| CD3+ HLA DR+ cells/µL  time point 3 | 0 | 6.000 | 403.167 | 231.205 | 160.532 | 645.801 | 0.683 |
|  | 1 | 3.000 | 342.333 | 93.565 | 109.906 | 574.761 |  |
|  | all | 9.000 | 382.889 | 191.112 | 235.988 | 529.790 |  |
| Ratio CD4/CD8  time point 3 | 0 | 6.000 | 1.121 | 1.035 | 0.035 | 2.208 | 0.547 |
|  | 1 | 3.000 | 0.724 | 0.279 | 0.030 | 1.418 |  |
|  | all | 9.000 | 0.989 | 0.854 | 0.333 | 1.645 |  |
| T-cells CD3 %  time point 5 | 0 | 6.000 | 76.210 | 23.395 | 51.659 | 100.762 | 0.511 |
|  | 1 | 4.000 | 67.044 | 14.988 | 43.194 | 90.893 |  |
|  | all | 10.000 | 72.544 | 20.034 | 58.212 | 86.875 |  |
| T-cells CD3 cells/µL  time point 5 | 0 | 6.000 | 1466.167 | 1030.422 | 384.805 | 2547.528 | 0.373 |
|  | 1 | 4.000 | 955.750 | 315.995 | 452.931 | 1458.569 |  |
|  | all | 10.000 | 1262.000 | 832.244 | 666.649 | 1857.351 |  |
| T-help CD3+ CD4+ %  time point 5 | 0 | 6.000 | 29.888 | 17.698 | 11.316 | 48.461 | 0.902 |
|  | 1 | 4.000 | 31.196 | 12.721 | 10.955 | 51.437 |  |
|  | all | 10.000 | 30.411 | 15.113 | 19.600 | 41.222 |  |
| T-help CD3+ CD4+ cells/µL  time point 5 | 0 | 6.000 | 557.167 | 317.153 | 224.335 | 889.999 | 0.680 |
|  | 1 | 4.000 | 471.250 | 301.285 | -8.162 | 950.662 |  |
|  | all | 10.000 | 522.800 | 296.829 | 310.462 | 735.138 |  |
| T-supp CD3+ CD8+ %  time point 5 | 0 | 6.000 | 46.123 | 29.296 | 15.378 | 76.868 | 0.465 |
|  | 1 | 4.000 | 34.181 | 11.064 | 16.576 | 51.786 |  |
|  | all | 10.000 | 41.346 | 23.572 | 24.484 | 58.209 |  |
| T-supp CD3+ CD8+ cells/µL  time point 5 | 0 | 6.000 | 906.500 | 907.755 | -46.131 | 1859.131 | 0.365 |
|  | 1 | 4.000 | 461.250 | 55.638 | 372.718 | 549.782 |  |
|  | all | 10.000 | 728.400 | 715.323 | 216.689 | 1240.111 |  |
| B-cells CD19 %  time point 5 | 0 | 6.000 | 14.284 | 21.671 | -8.458 | 37.027 | 0.531 |
|  | 1 | 4.000 | 21.937 | 9.480 | 6.853 | 37.022 |  |
|  | all | 10.000 | 17.345 | 17.507 | 4.822 | 29.869 |  |
| B-cells CD19 cells/µL  time point 5 | 0 | 6.000 | 485.333 | 979.771 | -542.874 | 1513.540 | 0.769 |
|  | 1 | 4.000 | 332.000 | 175.281 | 53.089 | 610.911 |  |
|  | all | 10.000 | 424.000 | 741.497 | -106.435 | 954.435 |  |
| NK-cells CD3 - 16+ 56 + %  time point 5 | 0 | 6.000 | 8.528 | 5.491 | 2.766 | 14.291 | 0.734 |
|  | 1 | 4.000 | 9.754 | 5.238 | 1.419 | 18.088 |  |
|  | all | 10.000 | 9.019 | 5.128 | 5.350 | 12.687 |  |
| NK-cells CD3 - 16+ 56 + cells/µL  time point 5 | 0 | 6.000 | 167.333 | 124.322 | 36.866 | 297.801 | 0.840 |
|  | 1 | 4.000 | 152.250 | 87.926 | 12.341 | 292.159 |  |
|  | all | 10.000 | 161.300 | 105.944 | 85.512 | 237.088 |  |
| CD3+ HLA DR+ %  time point 5 | 0 | 6.000 | 21.211 | 20.087 | 0.131 | 42.290 | 0.772 |
|  | 1 | 4.000 | 25.110 | 20.314 | -7.213 | 57.433 |  |
|  | all | 10.000 | 22.770 | 19.125 | 9.089 | 36.451 |  |
| CD3+ HLA DR+ cells/µL  time point 5 | 0 | 6.000 | 308.167 | 176.914 | 122.507 | 493.827 | 0.928 |
|  | 1 | 4.000 | 298.500 | 128.043 | 94.755 | 502.245 |  |
|  | all | 10.000 | 304.300 | 151.255 | 196.099 | 412.501 |  |
| Ratio CD4/CD8  time point 5 | 0 | 6.000 | 1.135 | 1.002 | 0.084 | 2.186 | 0.739 |
|  | 1 | 5.000 | 1.335 | 0.903 | 0.213 | 2.456 |  |
|  | all | 11.000 | 1.226 | 0.916 | 0.611 | 1.841 |  |
| T-cells CD3 %  time point 6 | 0 | 5.000 | 74.513 | 32.340 | 34.358 | 114.668 | 0.343 |
|  | 1 | 4.000 | 57.740 | 4.078 | 51.250 | 64.229 |  |
|  | all | 9.000 | 67.058 | 24.644 | 48.115 | 86.001 |  |
| T-cells CD3 cells/µL  time point 6 | 0 | 5.000 | 2010.600 | 957.872 | 821.244 | 3199.956 | 0.049 |
|  | 1 | 4.000 | 822.000 | 267.217 | 396.799 | 1247.201 |  |
|  | all | 9.000 | 1482.333 | 937.002 | 762.090 | 2202.577 |  |
| T-help CD3+ CD4+ %  time point 6 | 0 | 5.000 | 32.128 | 27.095 | -1.516 | 65.771 | 0.707 |
|  | 1 | 4.000 | 25.661 | 20.941 | -7.661 | 58.982 |  |
|  | all | 9.000 | 29.253 | 23.305 | 11.339 | 47.167 |  |
| T-help CD3+ CD4+ cells/µL  time point 6 | 0 | 5.000 | 781.600 | 477.949 | 188.148 | 1375.052 | 0.188 |
|  | 1 | 4.000 | 383.250 | 287.230 | -73.797 | 840.297 |  |
|  | all | 9.000 | 604.556 | 435.010 | 270.177 | 938.934 |  |
| T-supp CD3+ CD8+ %  time point 6 | 0 | 5.000 | 43.344 | 35.460 | -0.685 | 87.374 | 0.261 |
|  | 1 | 4.000 | 19.655 | 16.340 | -6.345 | 45.655 |  |
|  | all | 9.000 | 32.816 | 29.744 | 9.952 | 55.679 |  |
| T-supp CD3+ CD8+ cells/µL  time point 6 | 0 | 5.000 | 1248.600 | 1119.231 | -141.109 | 2638.309 | 0.157 |
|  | 1 | 4.000 | 325.750 | 301.464 | -153.946 | 805.446 |  |
|  | all | 9.000 | 838.444 | 947.095 | 110.443 | 1566.446 |  |
| B-cells CD19 %  time point 6 | 0 | 5.000 | 18.290 | 31.298 | -20.572 | 57.152 | 0.498 |
|  | 1 | 4.000 | 29.764 | 5.824 | 20.497 | 39.030 |  |
|  | all | 9.000 | 23.389 | 23.218 | 5.543 | 41.236 |  |
| Antikörper / B-cells  time point 6 | 0 | 5.000 | 2.143 | 4.557 | -3.515 | 7.800 | 0.566 |
|  | 1 | 4.000 | 3.883 | 3.945 | -2.394 | 10.160 |  |
|  | all | 9.000 | 2.916 | 4.130 | -0.259 | 6.091 |  |
| NK-cells CD3 - 16+ 56 + %  time point 6 | 0 | 5.000 | 6.781 | 5.906 | -0.553 | 14.114 | 0.606 |
|  | 1 | 4.000 | 8.967 | 6.213 | -0.920 | 18.854 |  |
|  | all | 9.000 | 7.752 | 5.766 | 3.320 | 12.184 |  |
| NK-cells CD3 - 16+ 56 + cells/µL  time point 6 | 0 | 5.000 | 176.200 | 115.904 | 32.286 | 320.114 | 0.578 |
|  | 1 | 4.000 | 134.750 | 91.069 | -10.161 | 279.661 |  |
|  | all | 9.000 | 157.778 | 101.510 | 79.751 | 235.805 |  |
| CD3+ HLA DR+ %  time point 6 | 0 | 5.000 | 22.649 | 31.372 | -16.305 | 61.602 | 0.543 |
|  | 1 | 4.000 | 11.900 | 12.290 | -7.656 | 31.456 |  |
|  | all | 9.000 | 17.872 | 24.100 | -0.654 | 36.397 |  |
| CD3+ HLA DR+ cells/µL  time point 6 | 0 | 5.000 | 617.800 | 857.859 | -447.373 | 1682.973 | 0.362 |
|  | 1 | 4.000 | 186.500 | 183.144 | -104.923 | 477.923 |  |
|  | all | 9.000 | 426.111 | 657.428 | -79.233 | 931.455 |  |
| Ratio CD4/CD8  time point 6 | 0 | 5.000 | 1.392 | 1.285 | -0.203 | 2.988 | 0.621 |
|  | 1 | 3.000 | 2.027 | 2.244 | -3.547 | 7.602 |  |
|  | all | 8.000 | 1.631 | 1.578 | 0.311 | 2.950 |  |
| T-cells CD3 %  time point 8 | 0 | 5.000 | 81.478 | 16.481 | 61.014 | 101.942 | 0.032 |
|  | 1 | 2.000 | 43.124 | 11.543 | -60.582 | 146.830 |  |
|  | all | 7.000 | 70.520 | 23.527 | 48.760 | 92.279 |  |
| T-cells CD3 cells/µL  time point 8 | 0 | 5.000 | 1461.000 | 653.756 | 649.255 | 2272.745 | 0.614 |
|  | 1 | 2.000 | 1178.000 | 521.845 | -3510.590 | 5866.590 |  |
|  | all | 7.000 | 1380.143 | 591.090 | 833.476 | 1926.809 |  |
| T-help CD3+ CD4+ %  time point 8 | 0 | 5.000 | 38.954 | 23.298 | 10.026 | 67.881 | 0.380 |
|  | 1 | 2.000 | 22.046 | 5.856 | -30.565 | 74.656 |  |
|  | all | 7.000 | 34.123 | 20.872 | 14.819 | 53.426 |  |
| T-help CD3+ CD4+ cells/µL  time point 8 | 0 | 5.000 | 599.600 | 206.424 | 343.291 | 855.909 | 0.988 |
|  | 1 | 2.000 | 602.500 | 267.993 | -1805.326 | 3010.326 |  |
|  | all | 7.000 | 600.429 | 200.946 | 414.585 | 786.273 |  |
| T-supp CD3+ CD8+ %  time point 8 | 0 | 5.000 | 43.473 | 26.804 | 10.192 | 76.754 | 0.315 |
|  | 1 | 2.000 | 21.021 | 4.637 | -20.643 | 62.685 |  |
|  | all | 7.000 | 37.058 | 24.547 | 14.356 | 59.761 |  |
| T-supp CD3+ CD8+ cells/µL  time point 8 | 0 | 5.000 | 888.200 | 763.959 | -60.380 | 1836.780 | 0.624 |
|  | 1 | 2.000 | 584.500 | 283.550 | -1963.094 | 3132.094 |  |
|  | all | 7.000 | 801.429 | 651.497 | 198.894 | 1403.963 |  |
| B-cells CD19 %  time point 8 | 0 | 5.000 | 5.866 | 7.506 | -3.453 | 15.186 | 0.005 |
|  | 1 | 2.000 | 48.813 | 18.854 | -120.580 | 218.205 |  |
|  | all | 7.000 | 18.137 | 23.150 | -3.274 | 39.547 |  |
| B-cells CD19 cells/µL  time point 8 | 0 | 5.000 | 119.600 | 158.681 | -77.429 | 316.629 | 0.048 |
|  | 1 | 2.000 | 1654.500 | 1547.857 | -12252.441 | 15561.441 |  |
|  | all | 7.000 | 558.143 | 988.448 | -356.019 | 1472.305 |  |
| NK-cells CD3 - 16+ 56 + %  time point 8 | 0 | 5.000 | 11.928 | 8.423 | 1.470 | 22.386 | 0.517 |
|  | 1 | 2.000 | 7.271 | 6.014 | -46.763 | 61.304 |  |
|  | all | 7.000 | 10.597 | 7.648 | 3.525 | 17.670 |  |
| NK-cells CD3 - 16+ 56 + cells/µL  time point 8 | 0 | 5.000 | 213.800 | 177.154 | -6.166 | 433.766 | 0.162 |
|  | 1 | 2.000 | 764.500 | 823.779 | -6636.864 | 8165.864 |  |
|  | all | 7.000 | 371.143 | 454.128 | -48.855 | 791.141 |  |
| CD3+ HLA DR+ %  time point 8 | 0 | 5.000 | 15.266 | 6.240 | 7.518 | 23.015 | 0.308 |
|  | 1 | 2.000 | 9.446 | 5.650 | -41.315 | 60.207 |  |
|  | all | 7.000 | 13.603 | 6.273 | 7.802 | 19.405 |  |
| CD3+ HLA DR+ cells/µL  time point 8 | 0 | 5.000 | 280.800 | 167.522 | 72.794 | 488.806 | 0.684 |
|  | 1 | 2.000 | 226.500 | 20.506 | 42.260 | 410.740 |  |
|  | all | 7.000 | 265.286 | 139.575 | 136.200 | 394.371 |  |
| Ratio CD4/CD8  time point 8 | 0 | 5.000 | 1.415 | 1.140 | 0.000 | 2.830 | 0.681 |
|  | 1 | 2.000 | 1.044 | 0.049 | 0.605 | 1.482 |  |
|  | all | 7.000 | 1.309 | 0.948 | 0.432 | 2.186 |  |

Table S3: Antibodies used for immune profiling in this study.

| Antigen | Label | Ig-chain | Clone | Source |
| --- | --- | --- | --- | --- |
| Ig(G1) | PC5 | IgG1 | 679.1Mc7 | Beckman-Coulter A07798 |
| HLA-DR | PC7 | IgG1 | Immu-357 | Beckman-Coulter B49180 |
| CD45 | FITC | IgG2b | B3821F4A | Beckman-Coulter 6607073 |
| CD4 | RD1 | IgG1 | SFCI12T4D11 | Beckman-Coulter 6607073 |
| CD8 | ECD | IgG1 | SFCI21Thy2D3 | Beckman-Coulter 6607073 |
| CD3 | PC5 | IgG1 | UCHT1 | Beckman-Coulter 6607073 |
| CD56 | RD1 | IgG1 | N901/NKH-1 | Beckman-Coulter 6607073 |
| CD19 | ECD | IgG1 | J3-119 | Beckman-Coulter 6607073 |

Table S4: Mann-Whitney U test results for weak versus strong responders corresponding to Figure 3.

| **Ranks** | | | | | **Statistics** | | | | |
| --- | --- | --- | --- | --- | --- | --- | --- | --- | --- |
| Response Code  0: weak response; 1: good response | | N | Middle Rank | Ranksum | Mann-Whitney-U-Test | Wilcoxon-W | Z | Asymp. Sig. (2-sided) | Exact Sig. [2*(1-sided Sig.)] |
| SARS-CoV2- Antibodies  time point 1 | 0 | 7 | 7.50 | 52.50 |  |  |  |  |  |
|  | 1 | 8 | 8.44 | 67.50 |  |  |  |  |  |
|  | All | 15 |  |  | 24.500 | 52.500 | -0.935 | 0.350 | 0.694^b^ |
| T-cells CD3 %  time point 1 | 0 | 7 | 9.86 | 69.00 |  |  |  |  |  |
|  | 1 | 7 | 5.14 | 36.00 |  |  |  |  |  |
|  | All | 14 |  |  | 8.000 | 36.000 | -2.108 | 0.035 | 0.038^b^ |
| T-cells CD3 cells/µL  time point 1 | 0 | 7 | 7.71 | 54.00 |  |  |  |  |  |
|  | 1 | 7 | 7.29 | 51.00 |  |  |  |  |  |
|  | All | 14 |  |  | 23.000 | 51.000 | -0.192 | 0.848 | 0.902^b^ |
| T-help CD3+ CD4+ %  time point 1 | 0 | 7 | 5.86 | 41.00 |  |  |  |  |  |
|  | 1 | 7 | 9.14 | 64.00 |  |  |  |  |  |
|  | All | 14 |  |  | 13.000 | 41.000 | -1.469 | 0.142 | 0.165^b^ |
| T-help CD3+ CD4+ cells/µL  time point 1 | 0 | 7 | 6.57 | 46.00 |  |  |  |  |  |
|  | 1 | 7 | 8.43 | 59.00 |  |  |  |  |  |
|  | All | 14 |  |  | 18.000 | 46.000 | -0.831 | 0.406 | 0.456^b^ |
| T-supp CD3+ CD8+ %  time point 1 | 0 | 7 | 9.14 | 64.00 |  |  |  |  |  |
|  | 1 | 7 | 5.86 | 41.00 |  |  |  |  |  |
|  | All | 14 |  |  | 13.000 | 41.000 | -1.469 | 0.142 | 0.165^b^ |
| T-supp CD3+ CD8+ cells/µL  time point 1 | 0 | 7 | 9.14 | 64.00 |  |  |  |  |  |
|  | 1 | 7 | 5.86 | 41.00 |  |  |  |  |  |
|  | All | 14 |  |  | 13.000 | 41.000 | -1.469 | 0.142 | 0.165^b^ |
| B-cells CD19 %  time point 1 | 0 | 7 | 5.00 | 35.00 |  |  |  |  |  |
|  | 1 | 7 | 10.00 | 70.00 |  |  |  |  |  |
|  | All | 14 |  |  | 7.000 | 35.000 | -2.236 | 0.025 | 0.026^b^ |
| B-cells CD19 cells/µL  time point 1 | 0 | 7 | 5.00 | 35.00 |  |  |  |  |  |
|  | 1 | 7 | 10.00 | 70.00 |  |  |  |  |  |
|  | All | 14 |  |  | 7.000 | 35.000 | -2.236 | 0.025 | 0.026^b^ |
| NK-cells CD3 - 16+ 56 + %  time point 1 | 0 | 7 | 5.86 | 41.00 |  |  |  |  |  |
|  | 1 | 7 | 9.14 | 64.00 |  |  |  |  |  |
|  | All | 14 |  |  | 13.000 | 41.000 | -1.469 | 0.142 | 0.165^b^ |
| NK-cells CD3 - 16+ 56 + cells/µL  time point 1 | 0 | 7 | 6.43 | 45.00 |  |  |  |  |  |
|  | 1 | 7 | 8.57 | 60.00 |  |  |  |  |  |
|  | All | 14 |  |  | 17.000 | 45.000 | -0.958 | 0.338 | 0.383^b^ |
| CD3+ HLA DR+ %  time point 1 | 0 | 7 | 9.29 | 65.00 |  |  |  |  |  |
|  | 1 | 7 | 5.71 | 40.00 |  |  |  |  |  |
|  | All | 14 |  |  | 12.000 | 40.000 | -1.597 | 0.110 | 0.128^b^ |
| CD3+ HLA DR+ cells/µL  time point 1 | 0 | 7 | 9.86 | 69.00 |  |  |  |  |  |
|  | 1 | 7 | 5.14 | 36.00 |  |  |  |  |  |
|  | All | 14 |  |  | 8.000 | 36.000 | -2.108 | 0.035 | 0.038^b^ |
| Ratio CD4/CD8  time point 1 | 0 | 7 | 5.71 | 40.00 |  |  |  |  |  |
|  | 1 | 7 | 9.29 | 65.00 |  |  |  |  |  |
|  | All | 14 |  |  | 12.000 | 40.000 | -1.597 | 0.110 | 0.128^b^ |
|  |  |  |  |  |  |  |  |  |  |
| Diagnosis  1: Myeloma, 2: Lymphoma |  | N | Middle Rank | Ranksum | Mann-Whitney-U-Test | Wilcoxon-W | Z | Asymp. Sig. (2-sided) | Exact Sig. [2*(1-sided Sig.)] |
| SARS-CoV2- Antibodies  time point 1 | 1 | 8 | 7.31 | 58.50 |  |  |  |  |  |
|  | 2 | 5 | 6.50 | 32.50 |  |  |  |  |  |
|  | All | 13 |  |  | 17.500 | 32.500 | -0.791 | 0.429 | 0.724^b^ |
| T-cells CD3 %  time point 1 | 1 | 7 | 6.29 | 44 |  |  |  |  |  |
|  | 2 | 5 | 6.80 | 34 |  |  |  |  |  |
|  | All | 12 |  |  | 16.000 | 44.000 | -0.244 | 0.808 | 0.876^b^ |
| T-cells CD3 cells/µL  time point 1 | 1 | 7 | 5.43 | 38 |  |  |  |  |  |
|  | 2 | 5 | 8 | 40 |  |  |  |  |  |
|  | All | 12 |  |  | 10.000 | 38.000 | -1.218 | 0.223 | 0.268^b^ |
| T-help CD3+ CD4+ %  time point 1 | 1 | 7 | 5.71 | 40 |  |  |  |  |  |
|  | 2 | 5 | 7.60 | 38 |  |  |  |  |  |
|  | All | 12 |  |  | 12.000 | 40.000 | -0.893 | 0.372 | 0.432^b^ |
| T-help CD3+ CD4+ cells/µL  time point 1 | 1 | 7 | 4.86 | 34 |  |  |  |  |  |
|  | 2 | 5 | 8.80 | 44 |  |  |  |  |  |
|  | All | 12 |  |  | 6.000 | 34.000 | -1.868 | 0.062 | 0.073^b^ |
| T-supp CD3+ CD8+ %  time point 1 | 1 | 7 | 7.57 | 53 |  |  |  |  |  |
|  | 2 | 5 | 5 | 25 |  |  |  |  |  |
|  | All | 12 |  |  | 10.000 | 25.000 | -1.218 | 0.223 | 0.268^b^ |
| T-supp CD3+ CD8+ cells/µL  time point 1 | 1 | 7 | 6.14 | 43 |  |  |  |  |  |
|  | 2 | 5 | 7 | 35 |  |  |  |  |  |
|  | All | 12 |  |  | 15.000 | 43.000 | -0.406 | 0.685 | 0.755^b^ |
| B-cells CD19 %  time point 1 | 1 | 7 | 6.86 | 48 |  |  |  |  |  |
|  | 2 | 5 | 6 | 30 |  |  |  |  |  |
|  | All | 12 |  |  | 15.000 | 30.000 | -0.406 | 0.685 | 0.755^b^ |
| B-cells CD19 cells/µL  time point 1 | 1 | 7 | 6.57 | 46 |  |  |  |  |  |
|  | 2 | 5 | 6.40 | 32 |  |  |  |  |  |
|  | All | 12 |  |  | 17.000 | 32.000 | -0.081 | 0.935 | 1.000^b^ |
| NK-cells CD3 - 16+ 56 + %  time point 1 | 1 | 7 | 7 | 49 |  |  |  |  |  |
|  | 2 | 5 | 5.80 | 29 |  |  |  |  |  |
|  | All | 12 |  |  | 14.000 | 29.000 | -0.568 | 0.570 | 0.639^b^ |
| NK-cells CD3 - 16+ 56 + cells/µL  time point 1 | 1 | 7 | 5.71 | 40 |  |  |  |  |  |
|  | 2 | 5 | 7.60 | 38 |  |  |  |  |  |
|  | All | 12 |  |  | 12.000 | 40.000 | -0.893 | 0.372 | 0.432^b^ |
| CD3+ HLA DR+ %  time point 1 | 1 | 7 | 8.29 | 58 |  |  |  |  |  |
|  | 2 | 5 | 4 | 20 |  |  |  |  |  |
|  | All | 12 |  |  | 5.000 | 20.000 | -2.030 | 0.042 | 0.048^b^ |
| CD3+ HLA DR+ cells/µL  time point 1 | 1 | 7 | 7.29 | 51 |  |  |  |  |  |
|  | 2 | 5 | 5.40 | 27 |  |  |  |  |  |
|  | All | 12 |  |  | 12.000 | 27.000 | -0.893 | 0.372 | 0.432^b^ |
| Ratio CD4/CD8  time point 1 | 1 | 7 | 5.57 | 39 |  |  |  |  |  |
|  | 2 | 5 | 7.80 | 39 |  |  |  |  |  |
|  | All | 12 |  |  | 11.000 | 39.000 | -1.056 | 0.291 | 0.343^b^ |

Note: b, Not corrected for bindings.

Table S5: Statistics for data shown in Fig. 4.

Table S5. Results of the Mann-Whitney-U test for good and weak responders separated for myeloma and lymphoma patients before vaccination, corresponding to Fig. 4.

| **Descriptive Statistics** | | | | | | | | | |
| --- | --- | --- | --- | --- | --- | --- | --- | --- | --- |
| Diagnosis: Multiple Myeloma | N | Average | Std.-Deviation | Minimum | Maximum | Percentiles | | | |
|  |  |  |  |  |  | 25. | | 50. (Median) | 75. |
| T-cells CD3 cells/µL  time point 1 | 7 | 930.571 | 684.9021 | 367.0 | 2268.0 | 406.000 | | 645.000 | 1340.000 |
| T-help CD3+ CD4+ cells/µL  time point 1 | 7 | 312.143 | 182.1331 | 102.0 | 592.0 | 190.000 | | 265.000 | 533.000 |
| T-supp CD3+ CD8+ cells/µL  time point 1 | 7 | 598.429 | 649.3673 | 175.0 | 2007.0 | 198.000 | | 353.000 | 746.000 |
| B-cells CD19 cells/µL  time point 1 | 7 | 168.871 | 143.1792 | 3.1 | 380.0 | 25.000 | | 141.000 | 328.000 |
| NK-cells CD3 - 16+ 56 + cells/µL  time point 1 | 7 | 179.000 | 82.4015 | 9.0 | 260.0 | 164.000 | | 192.000 | 236.000 |
| CD3+ HLA DR+ cells/µL  time point 1 | 7 | 372.571 | 325.8931 | 143.0 | 1076.0 | 171.000 | | 282.000 | 423.000 |
| Ratio CD4/CD8  time point 1 | 7 | 0.84400 | 0.536567 | 0.132 | 1.675 | 0.25000 | | 0.95900 | 1.20500 |
| Diagnosis lymphoma | N | Average | Std.-Deviation | Minimum | Maximum | Percentiles | | | |
|  |  |  |  |  |  | 25. | | 50. (Median) | 75. |
| T-cells CD3 cells/µL  time point 1 | 5 | 1283.400 | 624.9234 | 592.0 | 2302.0 | 849.500 | | 1163.000 | 1777.500 |
| T-help CD3+ CD4+ cells/µL  time point 1 | 5 | 632.600 | 268.4163 | 206.0 | 896.0 | 389.500 | | 679.000 | 852.500 |
| T-supp CD3+ CD8+ cells/µL  time point 1 | 5 | 653.000 | 636.7939 | 298.0 | 1787.0 | 311.500 | | 408.000 | 1117.000 |
| B-cells CD19 cells/µL  time point 1 | 5 | 465.800 | 823.9319 | 7.0 | 1925.0 | 17.500 | | 71.000 | 1111.500 |
| NK-cells CD3 - 16+ 56 + cells/µL  time point 1 | 5 | 242.400 | 157.4176 | 88.0 | 471.0 | 94.000 | | 262.000 | 381.000 |
| CD3+ HLA DR+ cells/µL  time point 1 | 5 | 200.000 | 91.7960 | 94.0 | 346.0 | 130.500 | | 192.000 | 273.500 |
| Ratio CD4/CD8  time point 1 | 5 | 1.56180 | 1.161674 | 0.321 | 2.754 | 0.41300 | | 1.51900 | 2.73200 |
| **Ranks** | | | | | **Statistical Results** | | | | |
| Multiple Myeloma | response > 500 | N | Middle rank | Rank Sum | Mann-Whitney-U-Test | Wilcoxon-W | Z | Asymp. Sig.  (2-sided) | Exact Sig.  [2*(1-sided Sig.)] |
| T-cells CD3 cells/µL  time point 1 | 0 = no | 3 | 4.00 | 12.00 |  |  |  |  |  |
|  | 1 = yes | 3 | 3.00 | 9.00 |  |  |  |  |  |
|  | Total | 6 |  |  | 3.000 | 9.000 | -0.655 | 0.513 | 0.700^b^ |
| T-help CD3+ CD4+ cells/µL  time point 1 | 0 | 3 | 3.33 | 10.00 |  |  |  |  |  |
|  | 1 | 3 | 3.67 | 11.00 |  |  |  |  |  |
|  | Total | 6 |  |  | 4.000 | 10.000 | -0.218 | 0.827 | 1.000^b^ |
| T-supp CD3+ CD8+ cells/µL  time point 1 | 0 | 3 | 4.33 | 13.00 |  |  |  |  |  |
|  | 1 | 3 | 2.67 | 8.00 |  |  |  |  |  |
|  | Total | 6 |  |  | 2.000 | 8.000 | -1.091 | 0.275 | 0.400^b^ |
| B-cells CD19 cells/µL  time point 1 | 0 | 3 | 2.00 | 6.00 |  |  |  |  |  |
|  | 1 | 3 | 5.00 | 15.00 |  |  |  |  |  |
|  | Total | 6 |  |  | 0.000 | 6.000 | -1.964 | 0.050 | 0.100^b^ |
| NK-cells CD3 - 16+ 56 + cells/µL  time point 1 | 0 | 3 | 3.00 | 9.00 |  |  |  |  |  |
|  | 1 | 3 | 4.00 | 12.00 |  |  |  |  |  |
|  | Total | 6 |  |  | 3.000 | 9.000 | -0.655 | 0.513 | 0.700^b^ |
| CD3+ HLA DR+ cells/µL  time point 1 | 0 | 3 | 4.67 | 14.00 |  |  |  |  |  |
|  | 1 | 3 | 2.33 | 7.00 |  |  |  |  |  |
|  | Total | 6 |  |  | 1.000 | 7.000 | -1.528 | 0.127 | 0.200^b^ |
| Ratio CD4/CD8  time point 1 | 0 | 3 | 3.00 | 9.00 |  |  |  |  |  |
|  | 1 | 3 | 4.00 | 12.00 |  |  |  |  |  |
|  | Total | 6 |  |  | 3.000 | 9.000 | -0.655 | 0.513 | 0.700^b^ |
| Lymphoma | response > 500 | N | middle Rank | Rank sum |  |  |  |  |  |
| T-cells CD3 cells/µL  time point 1 | 0 | 4 | 2.75 | 11.00 |  |  |  |  |  |
|  | 1 | 1 | 4.00 | 4.00 |  |  |  |  |  |
|  | Total | 5 |  |  | 1.000 | 11.000 | -0.707 | 0.480 | 0.800^b^ |
| T-help CD3+ CD4+ cells/µL  time point 1 | 0 | 4 | 2.50 | 10.00 |  |  |  |  |  |
|  | 1 | 1 | 5.00 | 5.00 |  |  |  |  |  |
|  | Total | 5 |  |  | 0.000 | 10.000 | -1.414 | 0.157 | 0.400^b^ |
| T-supp CD3+ CD8+ cells/µL  time point 1 | 0 | 4 | 3.25 | 13.00 |  |  |  |  |  |
|  | 1 | 1 | 2.00 | 2.00 |  |  |  |  |  |
|  | Total | 5 |  |  | 1.000 | 2.000 | -0.707 | 0.480 | 0.800^b^ |
| B-cells CD19 cells/µL  time point 1 | 00 | 4 | 2.75 | 11.00 |  |  |  |  |  |
|  | 1 | 1 | 4.00 | 4.00 |  |  |  |  |  |
|  | Total | 5 |  |  | 1.000 | 11.000 | -0.707 | 0.480 | 0.800^b^ |
| NK-cells CD3 - 16+ 56 + cells/µL  time point 1 | 0 | 4 | 3.00 | 12.00 |  |  |  |  |  |
|  |  | 1 | 3.00 | 3.00 |  |  |  |  |  |
|  | Total | 5 |  |  | 2.000 | 3.000 | 0.000 | 1.000 | 1.000^b^ |
| CD3+ HLA DR+ cells/µL  time point 1 | 0 | 4 | 3.50 | 14.00 |  |  |  |  |  |
|  | 1 | 1 | 1.00 | 1.00 |  |  |  |  |  |
|  | Total | 5 |  |  | 0.000 | 1.000 | -1.414 | 0.157 | 0.400^b^ |
| Ratio CD4/CD8  time point 1 | 0 | 4 | 2.50 | 10.00 |  |  |  |  |  |
|  | 1 | 1 | 5.00 | 5.00 |  |  |  |  |  |
|  | Total | 5 |  |  | 0.000 | 10.000 | -1.414 | 0.157 | 0.400^b^ |

Note: b, not corrected for bindings.


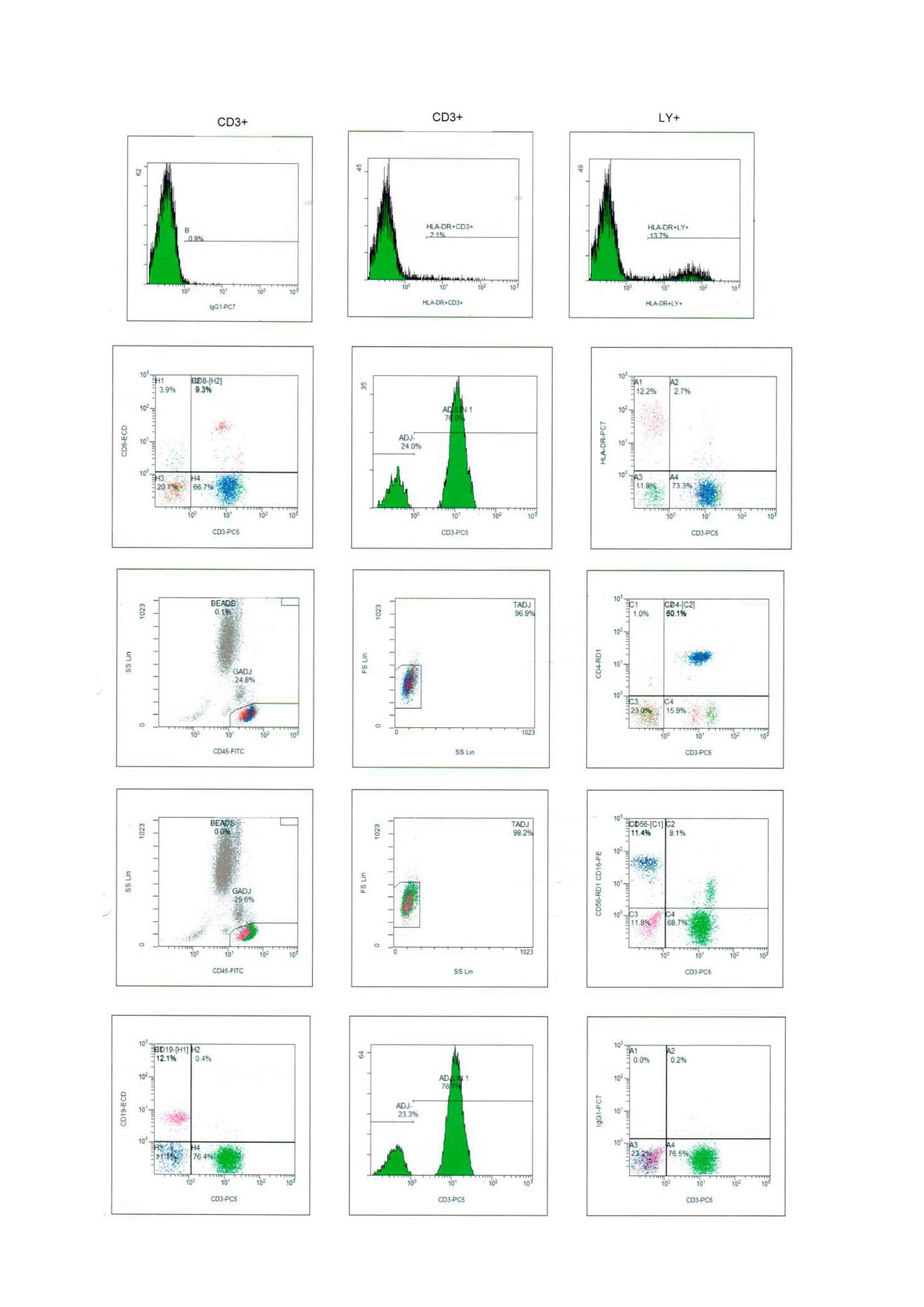


Figure S1: Representative example for immune profiling using the Beckman Coulter CYTO-STAT tetraCHROME panel, 6607073 as described in Table S3. LY: lymphocytes, ADJ: CD3- cells (B- and NK-cells), ADJLN1: CD3+ T-lymphocytes, GADJ: total number of lymphocytes in CD45/sideward scatter, TADJ: total number of vital cells.


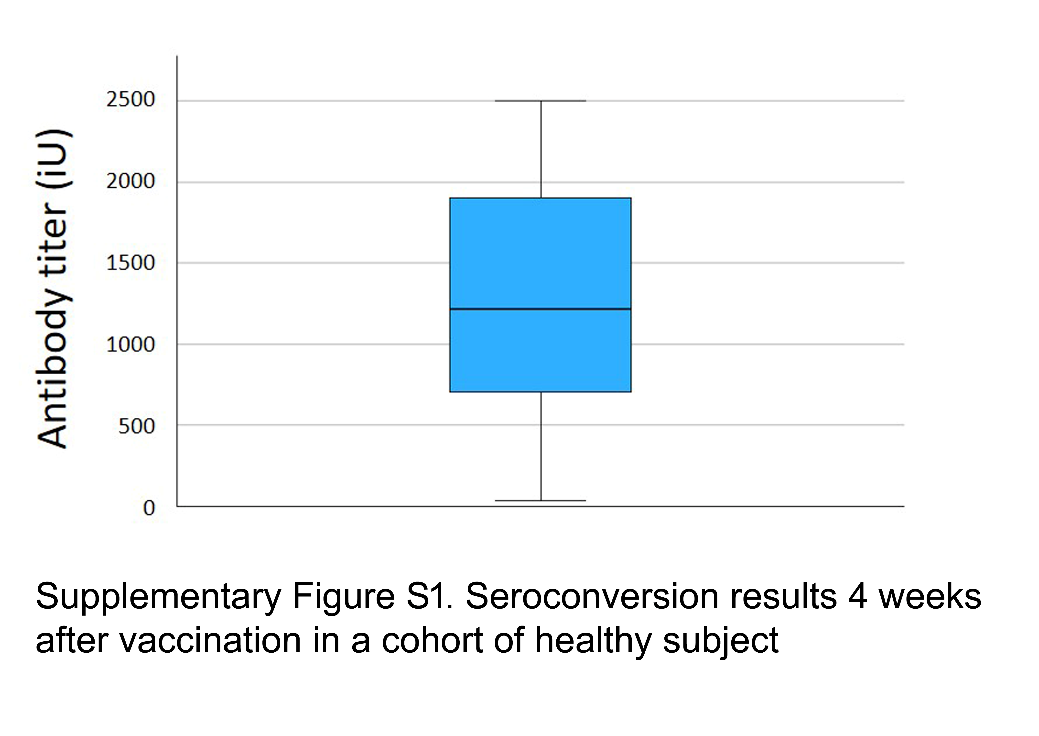


Figure S2: Seroconversion in a group of 300 healthy subjects from the second arm of this study 4 weeks after vaccination. Boxplot indicates median and quartiles of the antibody titer in these subjects.
