# Supplementary figures and images for: SARS-CoV-2 Vaccine-Induced Seroconversion and Immune Correlates in Patients with Hematological Malignancies. A Real World Study
Source: Oncol Res. 2025 Sep 26;33(10):2923–35. doi: 10.32604/or.2025.067561 (PMC12494110; doi:10.32604/or.2025.067561)

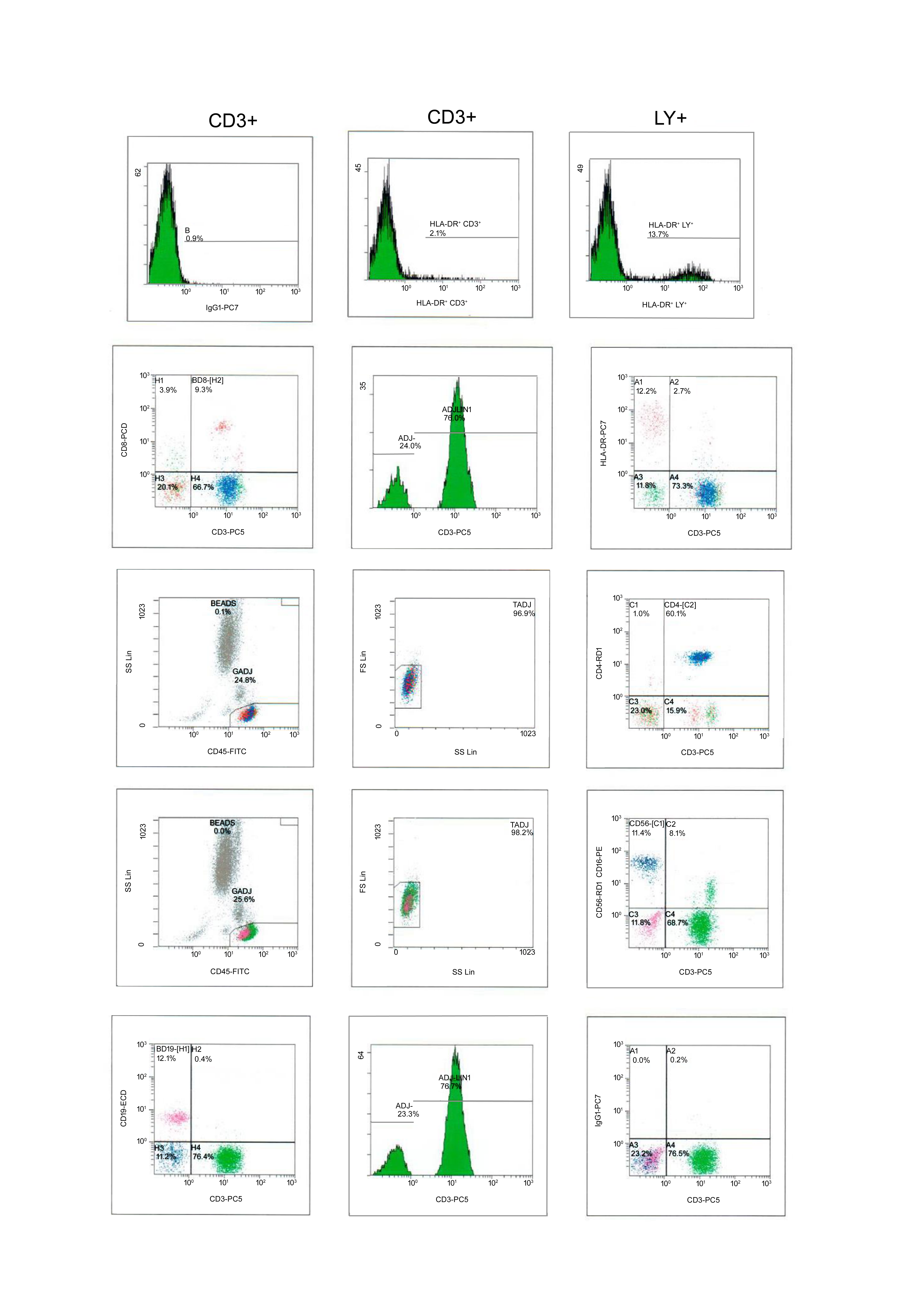

Supplement: Figure S1 [file OncolRes-33-67561-s002.tif]

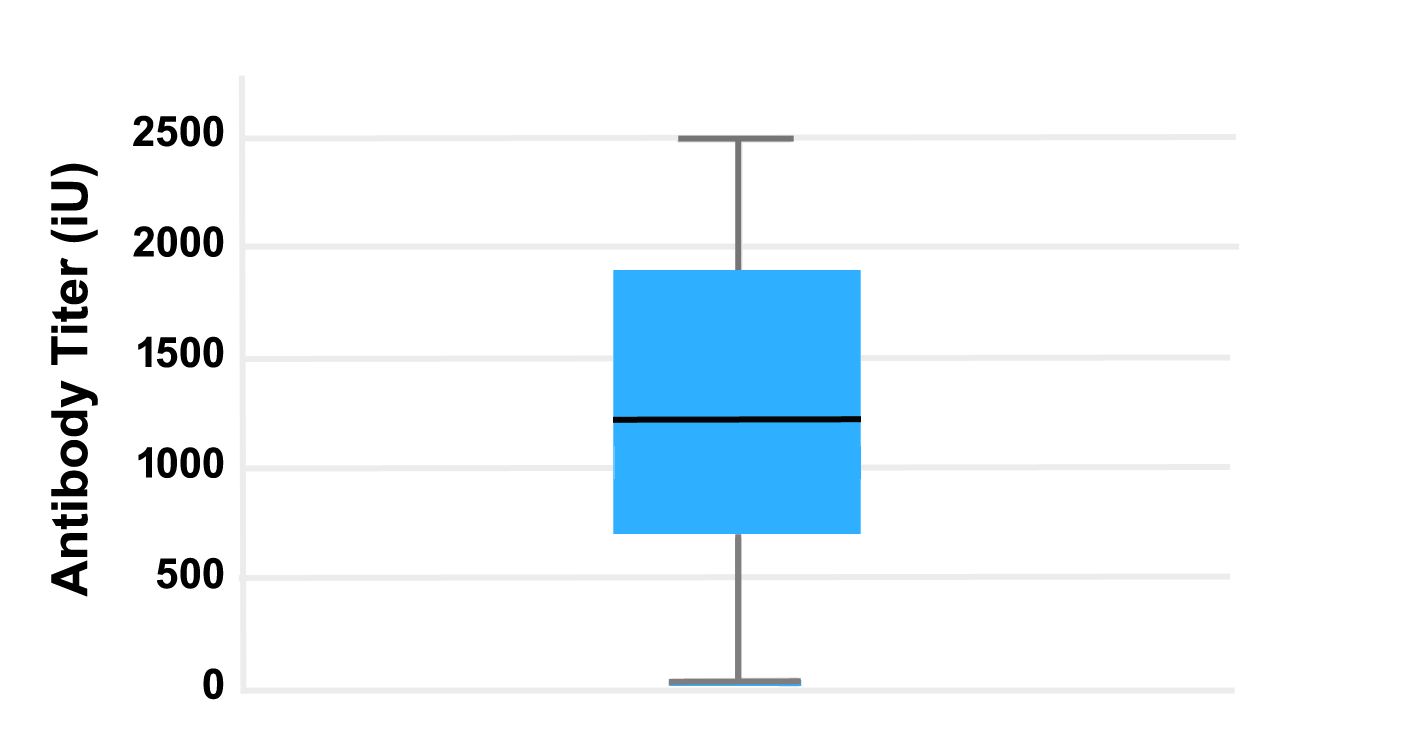

Supplement: Figure S2 [file OncolRes-33-67561-s003.tif]
